# Comparison of the Reactivity of PDMS with Terminal Allyl and Vinyl Groups in the Hydrosilylation Reaction

**DOI:** 10.3390/polym18192373

**Published:** 2026-09-29

**Authors:** Valentin Gubarev, Fedor Krylov, Sofia Ardabevskaia, Kseniya Bezlepkina, Kirill Bakanov, Sergey Milenin, Aziz Muzafarov

**Affiliations:** 1 Enikolopov Institute of Synthetic Polymeric Materials, Russian Academy of Sciences, Profsoyuznaya 70, 117393 Moscow, Russia; v.gubarev@ispm.ru (V.G.); krylov@ispm.ru (F.K.); ardabevskaya@ispm.ru (S.A.); bezlepkina_ka@ispm.ru (K.B.); bakanov@ispm.ru (K.B.); aziz@ispm.ru (A.M.); 2 Research Laboratory of New Silicone Materials and Technologies, Tula State Lev Tolstoy Pedagogical University, 300026 Tula, Russia

**Keywords:** polymers, oligomers, reactivity, hydrosilylation reaction, polydimethylsiloxanes, vinylsilanes, allylsilanes

## Abstract

Controlling the hydrosilylation rate during the curing of polydimethylsiloxanes (PDMS) is essential for regulating processing parameters and the properties of silicone materials. This study explores the possibility of achieving such control through differences in the reactivity of terminal allylsilyl and vinylsilyl groups. Difunctional PDMS bearing terminal allyl, vinyl, or hydride groups, together with a narrowly dispersed monofunctional PDMS bearing a terminal hydride group, were synthesized for model experiments. The starting compounds and hydrosilylation products were characterized by ^1^H and ^29^Si NMR spectroscopy and gel permeation chromatography. When allylsilyl and vinylsilyl groups were present in the same reaction mixture, hydrosilylation preferentially involved vinylsilyl groups. Additional model experiments confirmed the higher relative reactivity of vinylsilyl groups and revealed competing isomerization of the allylic double bond, which likely contributes to the slower hydrosilylation of allylsilyl groups. These findings indicate that differences in functional group reactivity can be used to control the sequence of hydrosilylation reactions and guide the molecular design of silicone materials with tailored properties.

## 1. Introduction

Linear polydimethylsiloxane oligomers (PDMS) with variable molecular weights have been key objects of polymer materials science for decades and are widely in demand in practical applications. They are used in both high-tech industrial and everyday applications, particularly in sealants [1], kitchen utensils [2], protective coatings [3], cosmetics [4], medicine, 3D printing [5], biomedical engineering [6,7], surface engineering [8], microfluidics [9,10], flexible electronics [11] and soft robotics [12]. The widespread use of polysiloxanes has been made possible by the combination of their elasticity, heat resistance, chemical inertness, hydrophobicity and biocompatibility [13,14]. The properties of PDMS-based materials directly depend on the molecular weight characteristics and functionality of the original polymers, which determine the crosslinking density and mechanical characteristics of the cured samples [15]. Moreover, the level of property control directly depends on the quality of the initial functional oligomers. The quality of linear PDMS telechelics with terminal functional groups, which are among the most sought-after products in block copolymer design, is primarily related to a narrow molecular weight distribution, the absence of low-molecular-weight impurities, and functional purity. Thus, in this context, it seems relevant to study the comparative reactivity of various functionalities in the composition of PDMS oligomers.

The hydrosilylation reaction, which in its classical form is the interaction between hydridosilyl and vinylsilyl groups, has been widely used in silicone chemistry since the advent of the first platinum catalysts [16,17,18] and platinum-based catalytic systems [19].

It should be noted that a quantitative comparison of the kinetic parameters of different substrates in the hydrosilylation reaction does not always allow for a correct assessment of their relative reactivity. This is because the hydrosilylation reaction is a complex and multifactorial process. Thus, during the reaction, the catalytic system based on platinum complexes can undergo changes, and the composition of the catalytically active particles can depend not only on the nature of the initial catalyst but also on the structure of the reagents and the process conditions [20].

Currently, the industrial standard for the hydrosilylation reaction is Karstedt’s catalyst, which is characterized by high activity, selectivity and efficiency in the curing processes of silicone compositions [21]. In general, the main components of such formulations are polysiloxanes containing vinyl and hydride functional groups. The different activities of vinyl substituents, which determine the kinetics of the process and the structure of the final material, are due to their position in the polysiloxane macromolecule. For example, when carrying out a hydrosilylation reaction, the terminal vinyl groups in PDMS have a higher reactivity compared to the side groups due to limitations in chain mobility and increased steric hindrance [22].

The literature describes approaches to the targeted regulation of the composition and properties of PDMS-based composites based on differences in the reactivity of vinyl and other unsaturated groups depending on their chemical environment [23], the length of the polymer chain, steric hindrance [24], their position in the polymer chain [25], and their role in the hydrosilylation reaction [26]. In particular, this principle [22] underlies the technology of liquid silicone rubbers for high-temperature vulcanization and is also used in the development of low-modulus silicone rubbers, where the different reactivities of functional groups allow for fine-tuning of the network structure and, as a consequence, the mechanical properties of the material. At the same time, it should be noted that views regarding a universal series of reactivity of unsaturated substituents in hydrosilylation reactions are ambiguous. For example, O. B. Gorbatsevich showed that during the formation of hyperbranched structures, the relative reactivity can depend significantly on the nature of the unsaturated group and the specific conditions of the system: when moving from vinyl to allyl and then to undecyl fragments, the efficiency of the hydrosilylation reaction increases, which is explained by a decrease in side processes and more efficient formation of hyperbranched structures [27]. Later, using the example of monomeric diallylmethylvinylsilane and hexamethyltrisiloxane, it was shown that allyl groups undergo the hydrosilylation reaction more slowly than vinyl groups [28]. However, the conclusion reached in this work is controversial, since it is widely known that allylsilanes are more reactive than vinylsilanes in electrophilic addition reactions [29,30,31,32,33]. In a later study published in 2018, the authors reported conclusions that were inconsistent with the findings discussed above. The objective of that work was to investigate how replacing vinyl groups with allyl groups in a vinyl-functional silicone resin (ViSR) affects the hydrosilylation kinetics, curing rate, and the structure and properties of the resulting material. This substitution resulted in an approximately 30-fold increase in the curing rate via hydrosilylation. The authors attributed this effect to p–dπ conjugation in ViSR, which was proposed to decrease the electron density of the carbon–carbon double bond. According to their interpretation, such an interaction is absent in allyl-functional resins because the methylene spacer between the silicon atom and the double bond interrupts the conjugation [34]. However, quantum-chemical studies of organosilicon compounds indicate that the contribution of silicon 3d orbitals is relatively small, suggesting that the observed differences in reactivity are likely governed by a combination of factors rather than by p–dπ conjugation alone [35]. Furthermore, the authors of Ref. [34] did not specify the platinum catalyst used in their study, although the nature of the catalyst is known to have a substantial influence on the kinetics of hydrosilylation reactions. Such discrepancies in the literature data indicate the absence of a single universal reactivity scale and the need for careful interpretation of kinetic data.

In this work, the relative reactivity of the terminal vinylsilyl and allylsilyl groups in PDMS was assessed not by kinetic constants, but by the degrees of conversion of functional groups achieved under identical hydrosilylation reaction conditions. This reaction was chosen as a promising model process for the subsequent production of siloxane rubbers based on liquid compositions.

## 2. Materials and Methods

### 2.1. Materials

The reagents purchased from a commercial source were used directly without further drying or purification.

Hexamethylcyclotrisiloxane (D3), 95% pure, used to prepare α-dimethylbutyl-ꞷ-dimethyl-polydimethylsiloxane, was purchased from ABCR (Karlsruhe, Germany); octamethylcyclotetrasiloxane (D_4_), 98% pure, used to prepare α,ꞷ-bis(dimethylvinyl)-polydimethylsiloxane, α,ꞷ-bis(dimethyl)-polydimethylsiloxane, and α,ꞷ-bis(dimethylallyl)-polydimethylsiloxane, was purchased from ABCR; Karstedt’s catalyst was purchased from Sigma-Aldrich (St. Louis, MO, USA); dimethylchlorosilane, 95% pure, was purchased from Macklin (Shanghai, China); N-butyllithium (2.7 M solution in toluene) used in the preparation of α-dimethylbutyl-ꞷ-dimethyl-polydimethylsiloxane was purchased from Acros Organics (Geel, Belgium); tetrahydrofuran (THF) with a purity of 99.8% and hexane with a purity of 97.76% were purchased from Ruskhim (Moscow, Russia); sodium carbonate (Na_2_CO_3_), used in the preparation of α,ꞷ-bis(dimethylvinyl)-polydimethylsiloxane and α,ꞷ-bis(dimethyl)-polydimethylsiloxane, was purchased from Ruskhim; trifluoromethanesulfonic acid (CF_3_SO_3_H) with 99% purity, used in the preparation of α,ꞷ-bis(dimethylvinyl)-polydimethylsiloxane and α,ꞷ-bis(dimethyl)-polydimethylsiloxane, was purchased from Ruskhim; 1,1,3,3-tetramethyldisiloxane (TMDS) with 98% purity, used in the preparation of α,ꞷ-bis(dimethyl)-polydimethylsiloxane, was purchased from IPK Yumex LLC (Ufa, Russia); and 1,3-divinyl-1,1,3,3-tetramethyldisiloxane and 1,3-diallyl-1,1,3,3-tetramethyldisiloxane with 98% and 97% purity, respectively, were obtained by hydrolytic condensation of the corresponding alkoxysilanes at ISPM RAS.

### 2.2. Characterization

NMR spectra were recorded on a Bruker AV II-300 spectrometer (Bruker, Eltingen, Germany) with an operating frequency of 300 MHz for ^1^H nuclei, in solutions of CDCl_3_. ^1^H, ^13^C, and ^29^Si NMR spectra were recorded on a Bruker Avance AV-300 spectrometer (300 MHz for ^1^H; 77.5 MHz for ^13^C; 59.6 MHz for ^29^Si). The spectra were processed using MestReNova software (v. 12.0).

GPC/SEC analysis was performed on a chromatographic system comprising a LC-10ADvp high-pressure pump (Shimadzu, Kyoto, Japan), a Smartline RI 2300 refractometer, and a Jetstream 2 Plus thermostat (Knauer, Berlin, Germany). The temperature was 40 ± 0.1 °C, with toluene as the eluent at a flow rate of 1.0 mL/min. Columns (300 × 7.8 mm) with phenogel sorbent (Phenomenex, Torrance, CA, USA), 5 μm, and a pore size from 50 to 105 Å were used. Columns were calibrated relative to Agilent polystyrene standards (California, USA). Chromatogram processing and molecular weight parameter calculation were performed using MultiChrom software for Windows version 1.6 (Ampersand, Moscow, Russia). GPC results were obtained for the main peak.

### 2.3. Preparation of PDMS

Preparation of α-dimethylbutyl-ꞷ-dimethyl-polydimethylsiloxane (1p).

To a solution of 19.21 g (0.0863 mol) of hexamethylcyclotrisiloxane in 200 mL of anhydrous hexane, 4.57 mL of a 2.7 M solution (0.0123 mol) of n-butyllithium was added with stirring. After 20 h of stirring, 100 mL of anhydrous THF was added to the reaction system. Then, 1.23 g (0.013 mol) of 95% dimethylchlorosilane was added dropwise into the flask with vigorous stirring. The resulting oligomer was washed with water, and the organic layer was dried over sodium sulfate. The volatile products were distilled off under reduced pressure (*p* = 1 mbar, T = 100 °C). Finally, 16.56 g (yield 80.2%) of a colorless, transparent liquid was obtained.

Mn (GPC, toluene) = 2190 Da, Mw/Mn = 1.2 (relative to polystyrene standards).

M_NMR_ = 2000 g/mol.

^1^H NMR (300 MHz, CDCl_3_): δ 4.71 (s, 1H, Si-H), 1.34–1.31 (m, 4H, -C_2_H_4_-), 0.91–0.86 (t, 3H, -C_2_H_4_-CH_3_), 0.56–0.51 (m, 2H, -C_2_H_4_-CH_2_-Si), 0.08 (s, 6H, Si-CH_3_).

Preparation of α,ꞷ-bis(dimethyl)-polydimethylsiloxane (2p).

First, 36.85 g (0.275 mol) of 1,1,3,3-tetramethyldisiloxane, 222 g (0.750 mol) of octamethylcyclotetrasiloxane, and 0.25 g (0.1% by weight) of trifluoromethanesulfonic acid as a catalyst were mixed. The reaction mixture was stirred for 5 h at a temperature of 60 °C. The catalyst was neutralized by adding Na_2_CO_3_ in a toluene solution; then, the product was filtered to remove the salt, and the volatile products were distilled off under reduced pressure (*p* = 1 mbar, E = 120 °C). Finally, 212 g (yield 85%) of the product was obtained as a viscous, colorless, transparent liquid.

Mn (GPC, toluene): 2670 Da, Mw/Mn = 1.5 (relative to polystyrene standards).

M_NMR_ = 2630 g/mol.

^1^H NMR (300 MHz, CDCl_3_): δ 4.71 (m, 1H, Si-H), 0.08 (m, 3H, Si-CH_3_).

Preparation of α,ꞷ-bis(dimethylvinyl)-polydimethylsiloxane (3p).

First, 2.80 g (0.015 mol) of 1,3-divinyl-1,1,3,3-tetramethyldisiloxane, 11.1 g (0.037 mol) of octamethylcyclotetrasiloxane, and 0.015 g (0.1 wt%) of trifluoromethanesulfonic acid as a catalyst were mixed. The reaction mixture was stirred for 5 h at 60 °C. The catalyst was neutralized by adding Na_2_CO_3_ in toluene solution. The product was then filtered to remove the salt, and the volatile products were distilled off under reduced pressure (1 mbar, 120 °C). Finally, 12.7 g (yield 85%) of the product was obtained as a viscous, colorless, transparent liquid.

Mn (GPC, toluene): 2120 Da, Mw/Mn = 1.7 (relative to polystyrene standards).

M _NMR_ = 2050 g/mol.

^1^H NMR (300 MHz, CDCl_3_): δ 6.20–5.70 (Si-vinyl), 0.08 (m, 3H, Si-CH_3_).

Preparation of α,ꞷ-bis(dimethylallyl)-polydimethylsiloxane (4p).

A mixture of 1 g (4.65 mmol) of 1,3-diallyl-1,1,3,3-tetramethyldisiloxane, 5.346 g (0.018 mol) of octamethylcyclotetrasiloxane, and 0.006 g (0.1 wt%) of tetramethylammonium hydroxide as a catalyst was prepared. The reaction mixture was stirred for 5 h at 100 °C. The catalyst was decomposed at 150 °C, and the volatile products were distilled off under reduced pressure (0.6 mbar, 125 °C). The product was obtained with a yield of 3.81 g (63%) as a viscous, colorless, transparent liquid.

Mn (GPC, toluene): 2100 Da, Mw/Mn = 1.7 (relative to polystyrene standards).

M _NMR_ = 2100 g/mol.

^1^H NMR (300 MHz, CDCl_3_): δ 5.86–5.72 (m, 1H, CH-allyl), 4.90–4.83 (m, 2H, CH_2_-allyl), 1.59–1.55 (m, 2H, Si-CH_2_), 0.07 (m, 3H, Si-CH_3_).

### 2.4. Hydrosilylation Reaction

Procedure for carrying out hydrosilylation reaction A—hydrosilylation of 1 equiv of vinyl–Si (polymer 3p) + 1 equiv of allyl–Si (polymer 4p) with 2 equiv of H–Si (polymer 1p).

First, 0.390 g (0.19 mmol) of compound 4p, 0.400 g (0.19 mmol) of compound 3p, and 0.7612 g (0.38 mmol) of compound 1p were mixed. The resulting mixture was stirred under argon for 5 min. After this, a sample was taken for ^1^H NMR and GPC analyses. Karstedt’s catalyst (29 ppm platinum) was then added, and stirring of the reaction mixture was continued for 6 h. After this, a second sample was taken for ^1^H NMR and GPC analyses. The isolation step was not included in the experiment.

Procedure for carrying out hydrosilylation reaction B—hydrosilylation of 1 equiv of vinyl–Si (polymer 3p) + 1 equiv of allyl–Si (polymer 4p) with 1 equiv of H–Si (polymer 2p).

First, 0.488 g (0.24 mmol) of compound 4p, 0.500 g (0.24 mmol) of compound 3p, and 0.626 g (0.24 mmol) of compound 2p were mixed. The resulting mixture was stirred under argon for 5 min. After this, a sample was taken for ^1^H NMR and GPC analyses. Karstedt’s catalyst (27 ppm platinum) was then added, and stirring of the reaction mixture was continued for 6 h. After this, a sample was taken again for ^1^H NMR and GPC analyses. The isolation step was not included in the experiment.

Procedure for carrying out hydrosilylation reaction Bs—hydrosilylation of 1 equiv of vinyl–Si (polymer 3p) + 1 equiv of allyl–Si (polymer 4p) with 1 equiv of H–Si (polymer 2p).

First, 0.488 g (0.24 mmol) of compound 4p, 0.500 g (0.24 mmol) of compound 3p, and 0.626 g (0.24 mmol) of compound 2p were mixed. The resulting mixture was stirred under argon for 5 min. Speier’s catalyst (48 ppm platinum) was then added, and stirring of the reaction mixture was continued for 6 h. After this, a sample was taken for ^1^H NMR analysis. The isolation step was not included in the experiment.

Procedure for carrying out hydrosilylation reaction C—hydrosilylation of 1 equiv of vinyl–Si (polymer 3p) + 1 equiv of allyl–Si (polymer 4p) with 1.5 equiv of H–Si (polymer 2p).

First, 0.390 g (0.19 mmol) of compound 4p, 0.400 g (0.19 mmol) of compound 3p, and 0.751 g (0.29 mmol) of compound 2p were mixed. The resulting mixture was stirred under argon for 5 min. After this, a sample was taken for ^1^H NMR and GPC analyses. Karstedt’s catalyst (29 ppm platinum) was then added, and stirring of the reaction mixture was continued for 6 h. After this, a sample was taken again for ^1^H NMR and GPC analyses. The isolation step was not included in the experiment.

Procedure for carrying out hydrosilylation reaction D-vinyl—hydrosilylation of 1 equiv of vinyl–Si (polymer 3p) with 2 equiv of H–Si (polymer 1p).

First, 0.390 g (0.19 mmol) of compound 3p and 0.761 g (0.38 mmol) of compound 1p were mixed. The resulting mixture was stirred under argon for 5 min. After this, a sample was taken for ^1^H NMR analysis. Karstedt’s catalyst (15 ppm platinum) was then added, and stirring of the reaction mixture was continued for one minute before the mixture was transferred to an NMR tube. The reaction kinetics were subsequently monitored in the NMR tube without further stirring. The ^1^H NMR spectrum was recorded before catalyst addition, 5 min after catalyst addition, and at identical time intervals thereafter for both reactions.

Procedure for carrying out hydrosilylation reaction D-allyl—hydrosilylation of 1 equiv of allyl–Si (polymer 4p) with 2 equiv of H–Si (polymer 1p).

First, 0.390 g (0.19 mmol) of compound 4p and 0.743 g (0.37 mmol) of compound 1p were mixed. The resulting mixture was stirred under argon for 5 min. After this, a sample was taken for ^1^H NMR analysis. Karstedt’s catalyst (15 ppm platinum) was then added, and stirring of the reaction mixture was continued for one minute before the mixture was transferred to an NMR tube. The reaction kinetics were subsequently monitored in the NMR tube without further stirring. The ^1^H NMR spectrum was recorded before catalyst addition, 5 min after catalyst addition, and at identical time intervals thereafter for both reactions.

Procedure for carrying out hydrosilylation reaction E—hydrosilylation of 1 equiv of vinyl–Si (polymer 3p) + 1 equiv of allyl–Si (polymer 4p) with 2 equiv of H–Si (polymer 1p).

First, 0.35 g (0.14 mmol) of compound 5p, 0.264 g (0.14 mmol) of compound 3p, and 0.579 g (0.28 mmol) of compound 1p were mixed. The resulting mixture was stirred under argon for 5 min. After this, a sample was taken for ^1^H NMR and GPC analyses. Karstedt’s catalyst (2 ppm platinum) was then added, and stirring of the reaction mixture was continued for one minute before the mixture was transferred to an NMR tube. The reaction kinetics were subsequently monitored in the NMR tube without further stirring.

## 3. Results and Discussion

**Figure 1:**
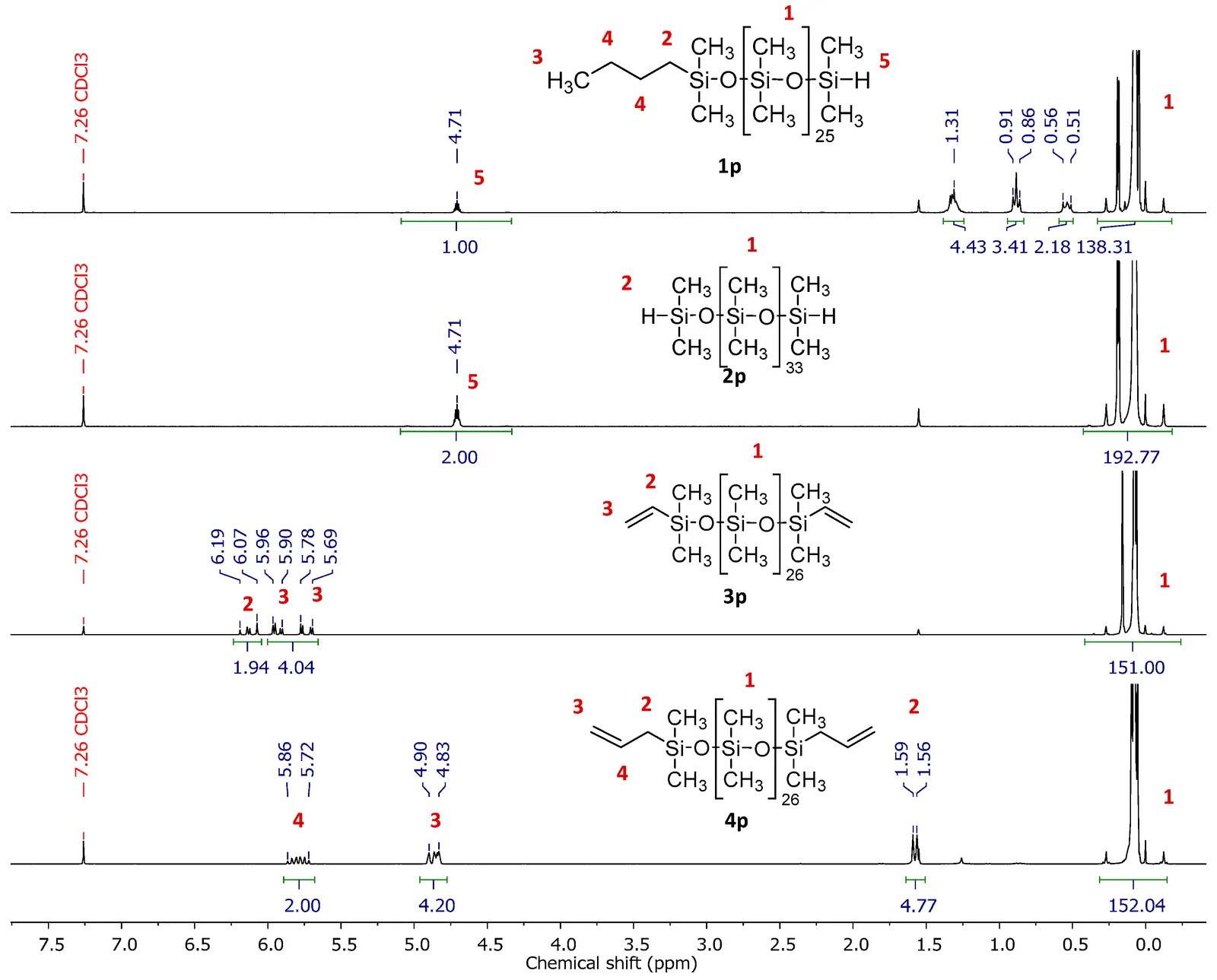
^1^H NMR spectrum of the initial oligomers.

To evaluate the different reactivities of allylsilyl and vinylsilyl end groups in PDMS with an oligomeric structure under hydrosilylation reaction conditions, two model reactions of the corresponding molecules with hydride-containing PDMS were carried out. The first reaction involved the interaction of two PDMS telechelics with terminal allyl and vinylsilyl groups, respectively, with a monofunctional hydride-containing polydimethylsiloxane and was intended to compare the reactivity of vinyl and allyl groups without significantly increasing the molecular weight of the products. The second reaction used PDMS with two terminal hydridosilyl groups to simulate the formation of high-molecular structures. To implement both model reactions, four initial siloxane oligomers (1p, 2p, 3p, 4p) with similar molecular weight characteristics were synthesized using the well-known ring-opening polymerization technique [36,37]. In particular, PDMS with terminal allyl groups was obtained according to a previously developed method of opening octamethyltetracyclosiloxane (D_4_) in the presence of tetramethylammonium hydroxide (TMAH) as an initiator and 1,1,3,3-tetramethyl-1,3-diallyldisiloxane as a chain terminator [38]. Although PDMS with terminal allyl groups can also be synthesized using trifluoromethanesulfonic acid, the use of TMAH provides closer agreement between experimentally determined and theoretical molecular weights. The obtained structures were fully characterized using ^1^H (Figure 1) and ^29^Si NMR spectroscopy, as well as gel permeation chromatography (GPC) (Figures S1–S8, Supporting Information). Table 1 presents the molecular weight characteristics of the obtained compounds.

To correctly compare the reactivity of PDMS telechelics containing vinyl and allyl end groups, samples were synthesized with the closest possible molecular weights (Table 1). This made it possible to compare systems that differ in the nature of the functional groups, eliminating the influence of chain length and associated conformational and diffusion factors that affect the rate of the hydrosilylation reaction.

**Scheme 1:**
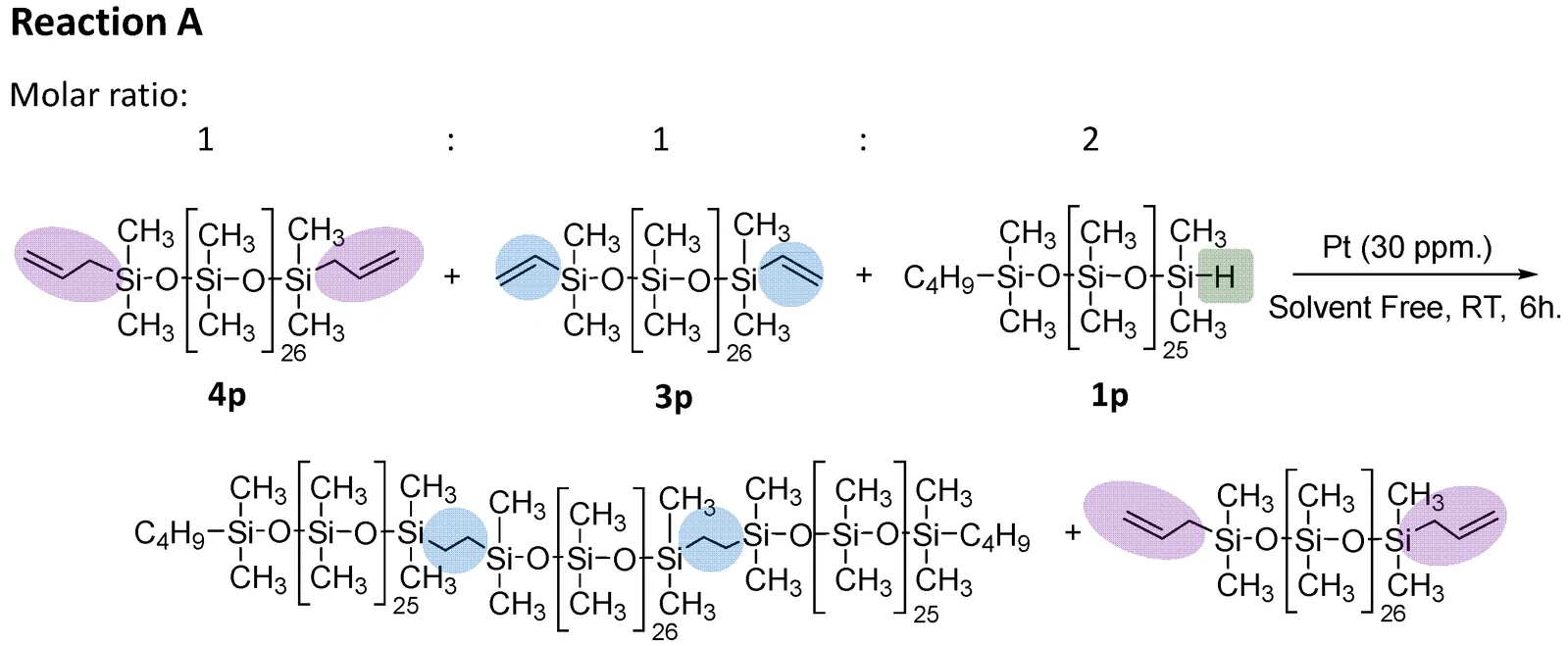
Model hydrosilylation reaction (reaction A). Hydrosilylation of 1 equiv of vinyl–Si (polymer **3p**) and 1 equiv of allyl–Si (polymer **4p**) with 2 equiv of H–Si (polymer **1p**). The functional groups are color-coded as follows: purple indicates the allyl group; blue denotes the vinyl group and the bond formed via the hydrosilylation of the hydride and vinyl groups; green represents the hydride.

The first model hydrosilylation reaction (reaction A) was carried out between compounds 4p, 3p, and 1p in the presence of Karstedt’s catalyst (30 ppm platinum) at room temperature, without solvents, for six hours under argon. The components were introduced into the reaction in a molar ratio of 4p, 3p, and 1p of 1:1:2, respectively (Table 2; Scheme 1). Table 2 shows the content of functional groups in the actual reaction mixture, calculated from NMR data, taking into account the associated measurement uncertainty. This observation is consistent with the overall conversion of unsaturated groups occasionally exceeding 100%.

An inert atmosphere was used to minimize potential side reactions associated with the presence of moisture and oxygen from the ambient air.

**Figure 2:**
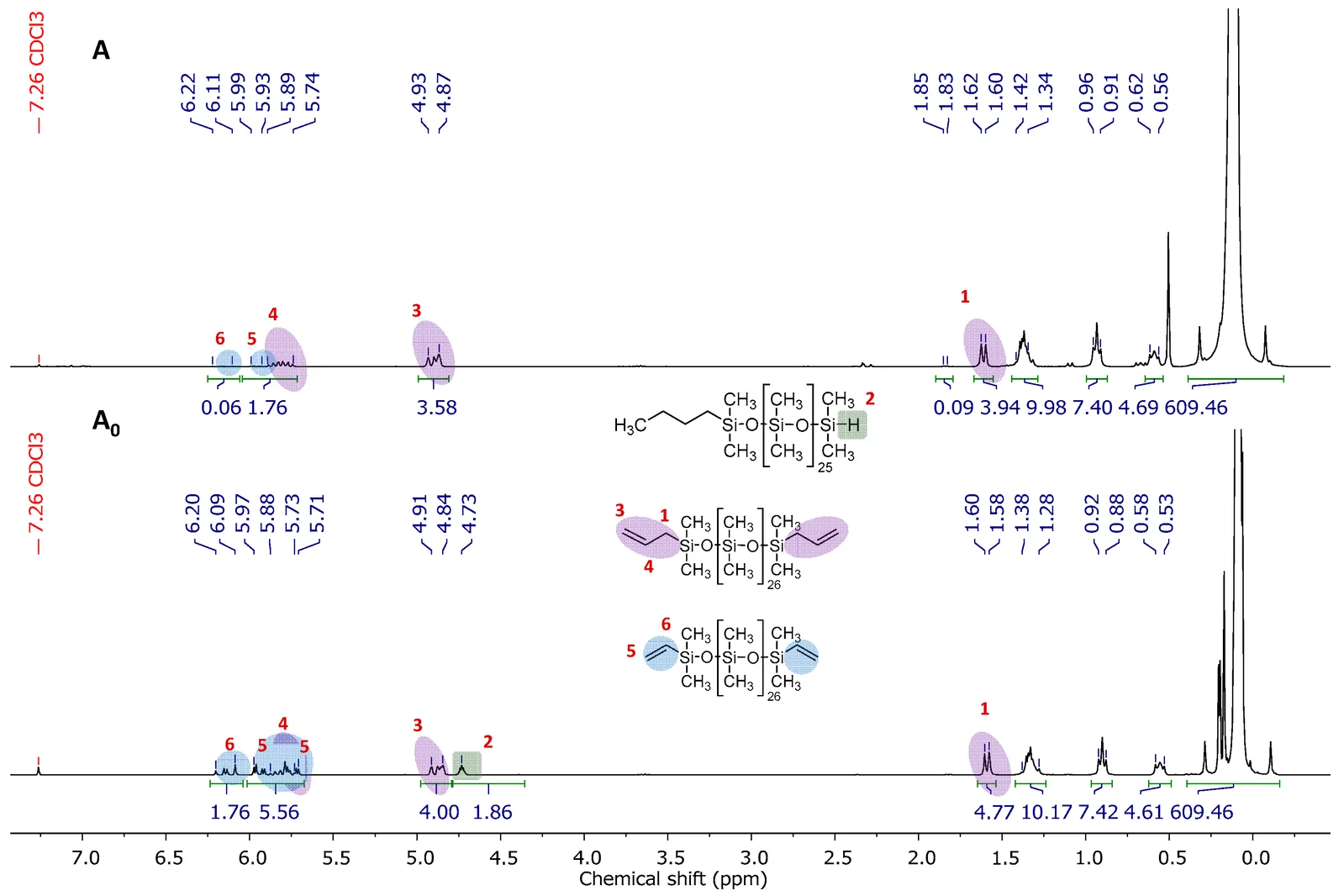
^1^H NMR spectrum of the reaction mixture before adding the catalyst (A_0_) and after the reaction (A). Colors denote the functional groups: purple—allyl group, blue—vinyl group, green—hydride.

To analyze the reaction progress using NMR, samples were taken from the reaction mixture before the addition of Karstedt’s catalyst (designated as A_0_) and after the reaction was completed (designated as A). This approach allows for the precise identification of all functional groups and their corresponding signals (Figure 2).

The reaction conversion was assessed based on the combined data from ^1^H and ^29^Si NMR spectra (Figures S9–S12, Supporting Information). Thus, based on the absence of signals from the protons of hydride groups (δ = 4.73 ppm) in the ^1^H NMR spectra and the signal from silicon associated with the hydride group (δ = −6.95 ppm) in the ^29^Si NMR spectra, one can judge the complete conversion of hydride groups during the process. In the ^1^H NMR spectra in the region of 5.97–5.71 ppm, an overlap of the protons of allyl and vinyl groups was observed; therefore, after equalizing the values of the integral intensities of the protons of the methyl groups at silicon before and after the reaction, the conversion was calculated by the ratio of the values of the integral intensities of the signals of -CH= protons in the vinyl group, designated by the number 6 in Figure 2, before and after the reaction. It was found that the vinyl groups had reacted almost completely, and the unreacted residue was 3.4%. The conversion of allyl groups was 10.5%. In the ^29^Si NMR spectra, signals of silicon associated with the allyl group (δ = 4.24 ppm), silicon corresponding to reaction products with vinyl groups (δ = 8.20, 7.95 ppm), and a small signal of silicon corresponding to reaction products with allyl groups (δ = 6.85 ppm) were observed (Figures S11 and S12, Supporting Information). Thus, it can be concluded that both hydrosilylation processes occur in the reaction system containing components with allyl and vinyl-functional groups, but the two processes proceed at substantially different rates, with hydrosilylation of the vinyl groups being significantly faster.

**Figure 3:**
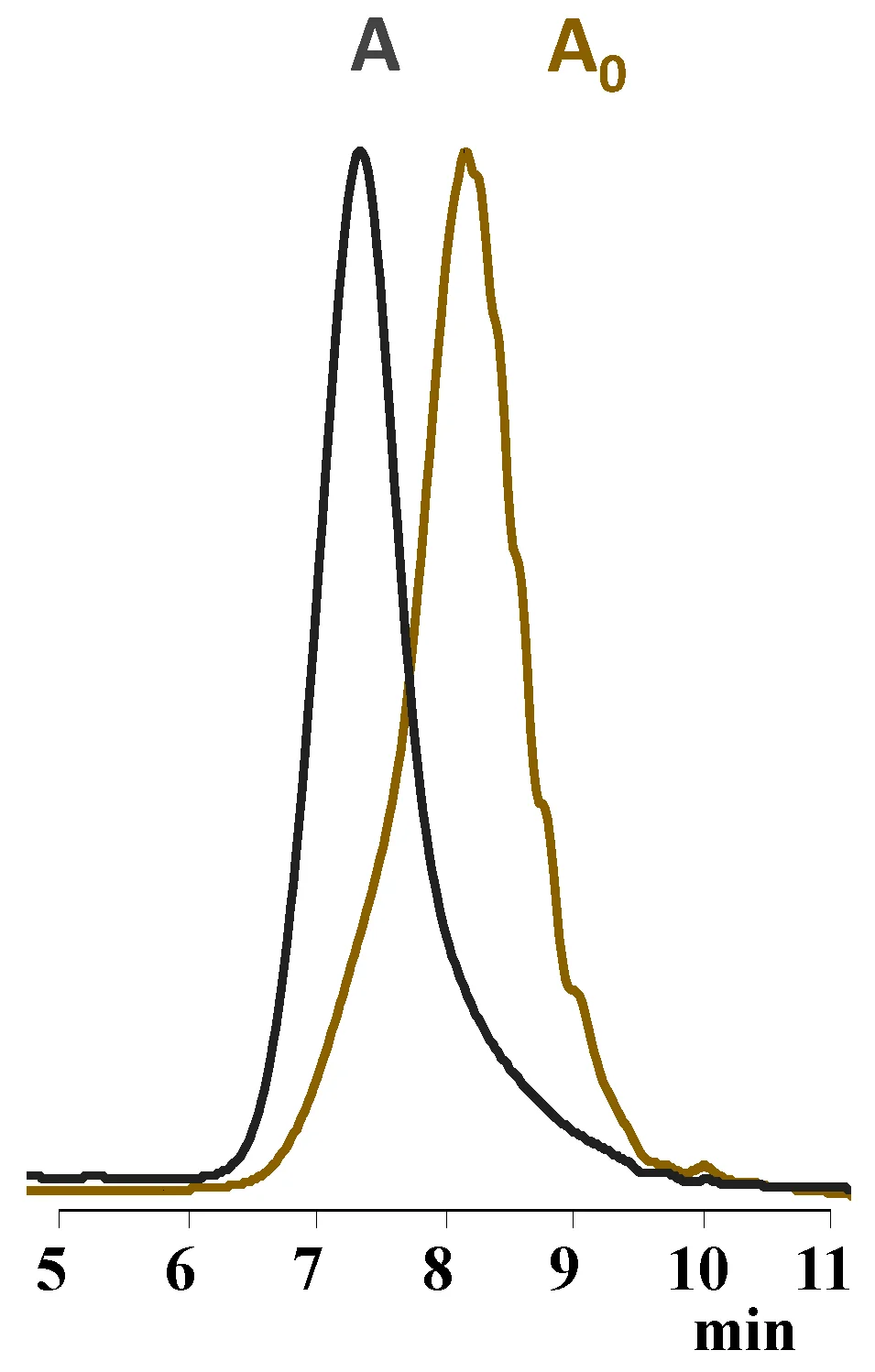
GPC curves of the reaction mixture before adding the catalyst (A_0_) and after the reaction was completed (A).

In addition, the process was also monitored using GPC. Figure 3 shows the curves for the initial mixture (curve A_0_) and the reaction products (curve A). A slight increase in the molecular weight of the product is observed, confirming the hydrosilylation reaction. The average molecular weight of the product (Mw) was 5600 Da.

**Scheme 2:**
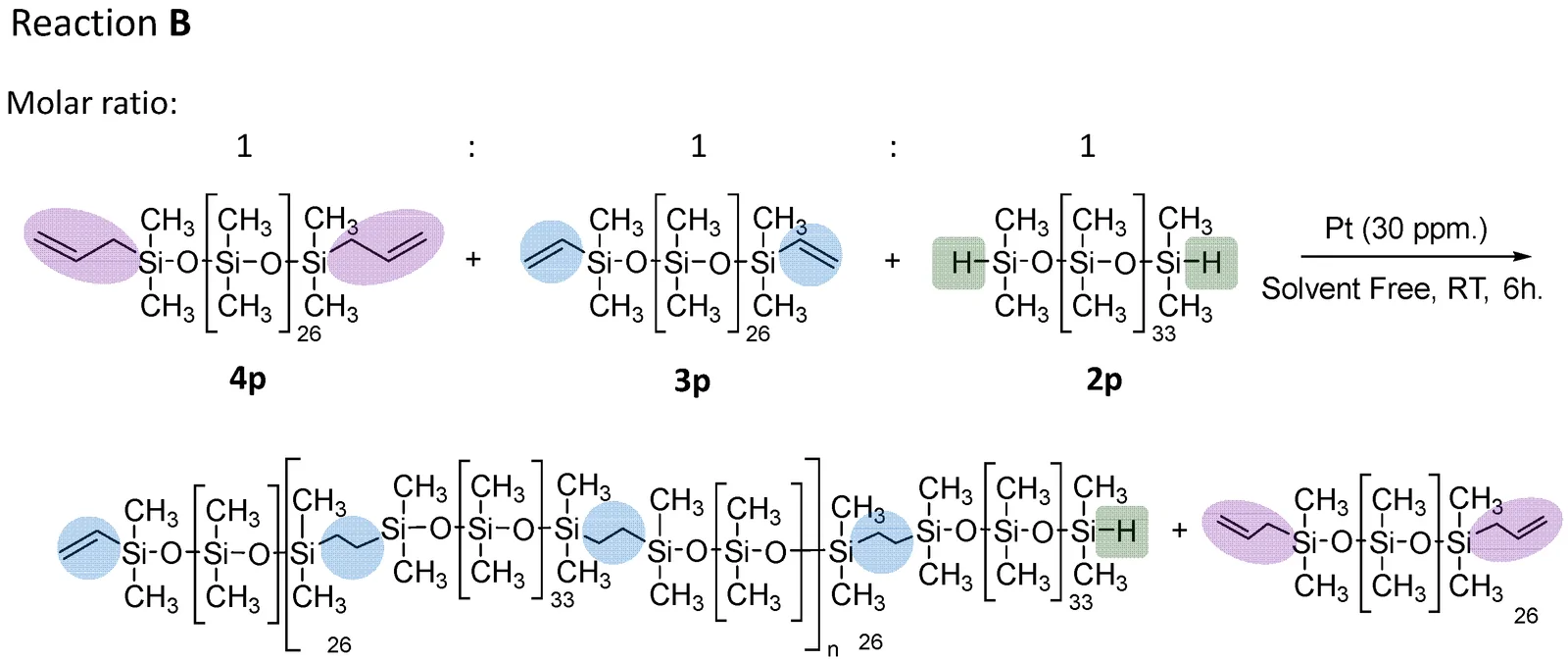
Model hydrosilylation reaction (reaction B)—hydrosilylation of 1 equiv of vinyl–Si (polymer 3p) + 1 equiv of allyl–Si (polymer 4p) with 1 equiv of H–Si (polymer 2p). Colors denote the functional groups: purple—allyl group, blue—vinyl group and the bond resulting from the hydride-vinyl hydrosilylation, green—hydride.

In the second part of the study, a PDMS oligomer containing two terminal Si-H bonds, 2p, was chosen as a model compound (Scheme 2). Thus, all reaction components were difunctional.

**Scheme 3:**
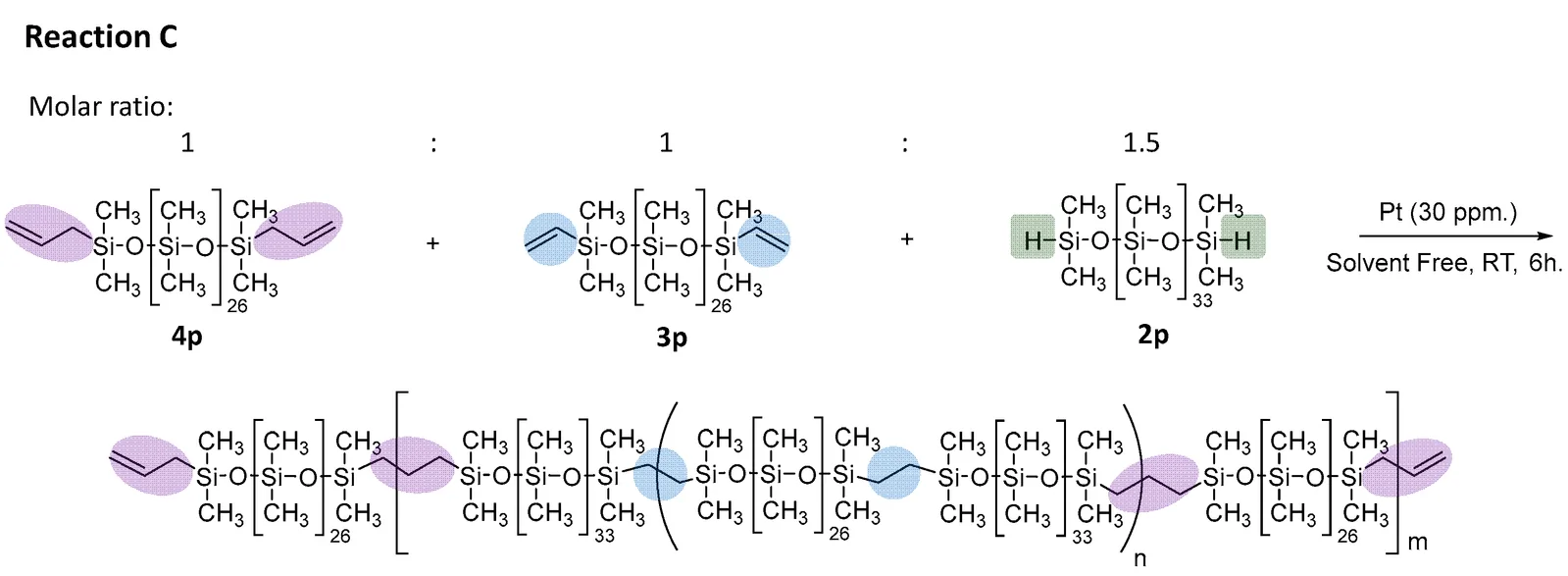
Model hydrosilylation reaction (reaction C)—hydrosilylation of 1 equiv of vinyl–Si (polymer 3p) + 1 equiv of allyl–Si (polymer 4p) with 1.5 equiv of H–Si (polymer 2p). Colors denote the functional groups: purple—allyl group, blue—vinyl group and the bond resulting from the hydride-vinyl hydrosilylation, green—hydride.

Two experiments were carried out: in the first case (Reaction B), the loadings of all components of the system were calculated in an equimolar ratio (Scheme 2); in the second case (Reaction C), component 2p was taken in 1.5-fold excess (Scheme 3). Excess hydride groups were introduced to achieve complete conversion of the vinyl groups—unlike the first experiment, where this was not achieved. The reactions were also carried out in the presence of Karstedt’s catalyst (30 ppm platinum) at room temperature and for 6 h under argon without the use of solvents.

**Figure 4:**
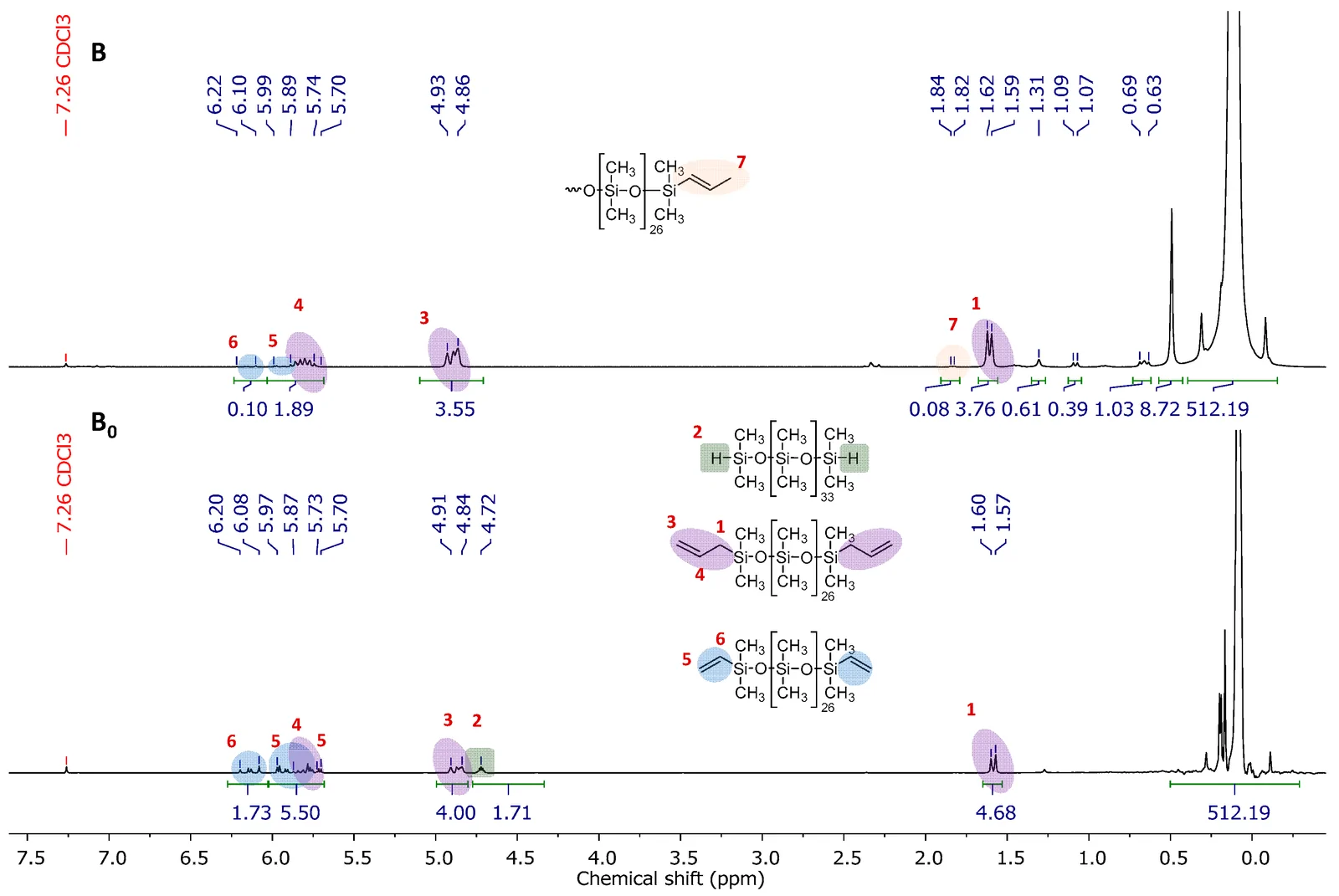
^1^H NMR spectrum of the reaction mixture before adding the catalyst (B_0_) and after the reaction was complete (B). Colors denote the functional groups: purple—allyl group, blue—vinyl group, green—hydride, peach—isomerized allyl group.

For reaction B, the conversion was estimated based on the combined data from ^1^H, ^29^Si and {^1^H, ^29^Si} HMBC (Figures S13–S17, Supporting Information) NMR spectra. Complete conversion was observed for the hydride groups, as there were no signals from the hydride group protons (δ = 4.72 ppm) in the ^1^H NMR spectra and no signal from the silicon associated with the hydride group (δ = −6.92 ppm) in the ^29^Si NMR spectra. In the ^1^H NMR spectra in the region δ = 5.97–5.70 ppm, the protons of the allyl and vinyl groups overlapped with each other, so the conversion of the vinyl groups was calculated similarly to the previous experiment (A). Analysis of the spectrum showed that the vinyl groups had reacted almost completely, with only 5.8% remaining unreacted (Figure 4). In contrast, the residue of the unreacted allyl groups was 88,7%. As in the previous reaction, signals corresponding to the protons of the isomerized allyl group were observed in the ^1^H NMR spectrum in the region of δ = 1.84–1.82 ppm, marked as 7.

In the ^29^Si NMR spectra, signals of silicon associated with the allyl group (δ = 4.35 ppm), silicon corresponding to reaction products with vinyl groups (δ = 8.26, 7.96 ppm), and a small signal of silicon corresponding to reaction products with allyl groups (δ = 6.91 ppm) were observed (Figures S17–S21, Supporting Information). The assignment of silicon signals was confirmed by {^1^H, ^29^Si} HMBC (Figure S17, Supporting Information).

**Figure 5:**
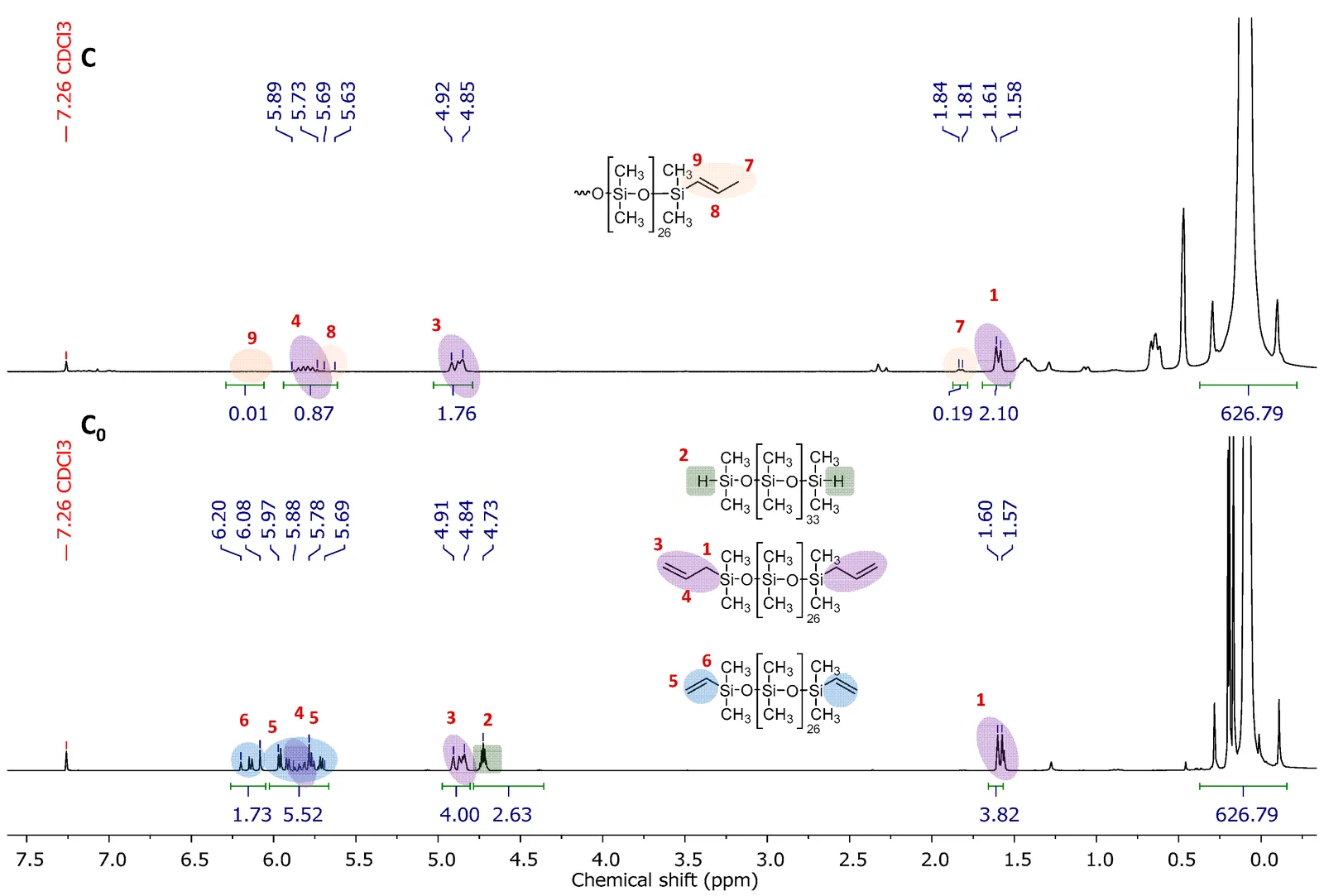
^1^H NMR spectrum of the reaction mixture before adding the catalyst (C_0_) and after the reaction was complete (C). Colors denote the functional groups: purple—allyl group, blue—vinyl group, green—hydride, peach—isomerized allyl group.

For reaction C, the reaction conversion was estimated based on the combined data from ^1^H and ^29^Si (Figures S18–S21, Supporting Information) NMR spectra. As in previous experiments, a complete absence of the signal of hydride group protons (δ = 4.73 ppm) in the ^1^H NMR spectra and the signal of silicon associated with the hydride group (δ = −6.95 ppm) in the ^29^Si NMR spectra was observed. In the ^1^H NMR spectra, the conversion of vinyl groups, estimated by the ratio of the integrated intensities of the -CH= proton signals in the vinyl group, designated by the number 6 in Figure 5 before and after the reaction, reached 100%, indicating their complete consumption. Although residual signals were observed in the corresponding spectral region, a more detailed analysis of the ^1^H NMR spectrum revealed that these signals originated from protons of isomerized allyl groups rather than unreacted vinyl groups. In addition, the signals corresponding to the protons of the isomerized allyl groups show higher intensity in the region of 1.84–1.81 ppm compared with those observed in reactions A and B (Figure 2 and Figure 4). This is most likely associated with the higher conversion of the allyl groups resulting from the increased hydride content in the initial reaction mixture. The isomerization of the allyl groups is investigated in greater detail below for reaction D-allyl. The residue of the unreacted allyl groups was 44%.

The ^29^Si NMR spectrum after the reaction also showed no signal from silicon with a vinyl group (δ = −4.14 ppm). Silicon signals corresponding to the reaction products with vinyl groups (δ = 8.30, 7.97 ppm) and reaction products with allyl groups (δ = 6.95 ppm) were observed (Figures S20 and S21, Supporting Information). Thus, it can be concluded that, in the reaction system containing components with allyl and vinyl groups, vinyl groups undergo hydrosilylation significantly faster than allyl groups.

**Figure 6:**
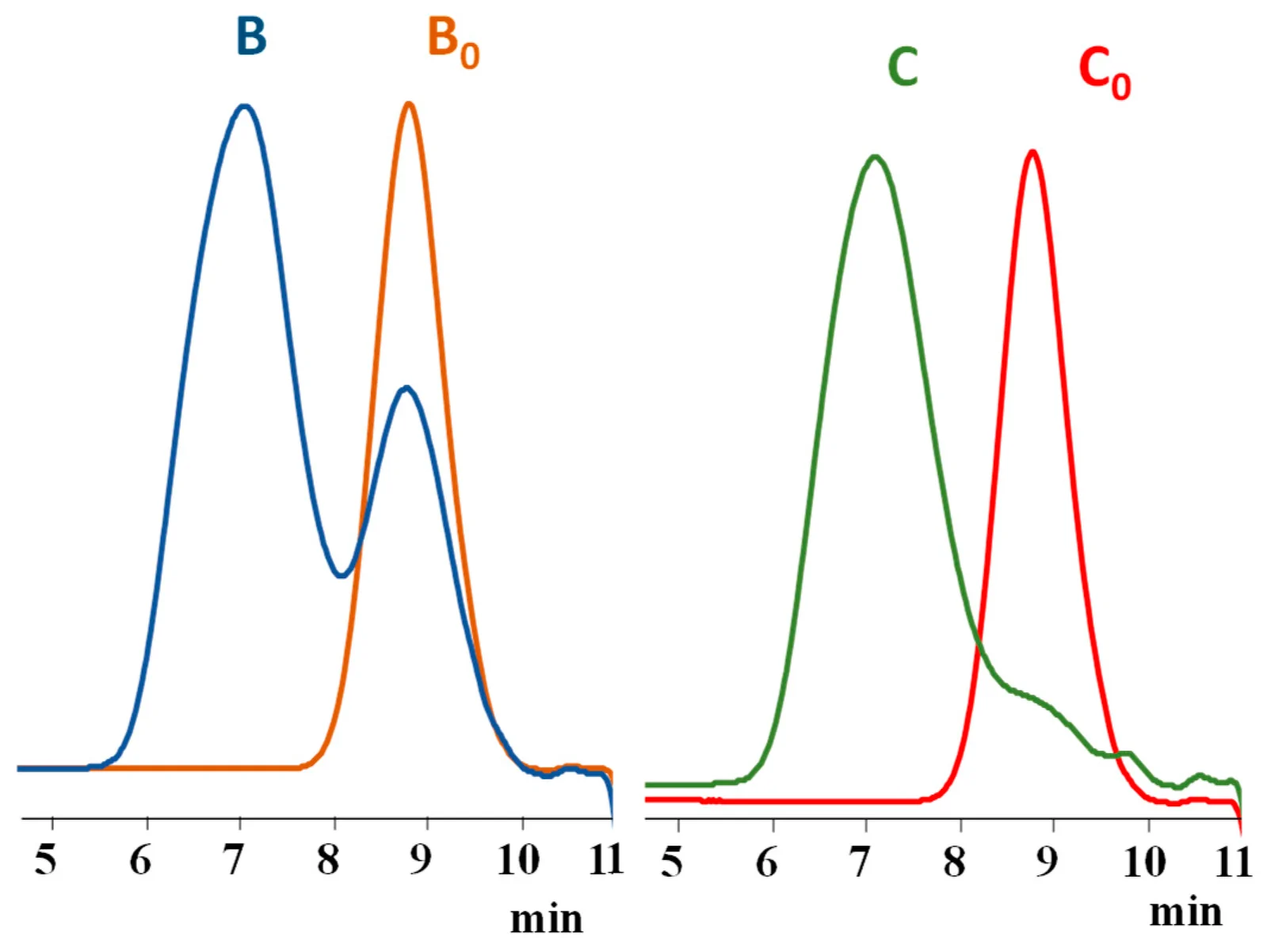
GPC curves of mixtures before adding the catalyst (B_0_, C_0_) and after the reactions had completely proceeded (B, C).

As in the previous case, the reaction products were analyzed by GPC; Figure 6 shows the curves of the reaction mixtures before the addition of Karstedt’s catalyst and after the completion of reactions B and C.

From the presented GPC curves, it is evident that in the case of reaction B, where the components were used in an equimolar ratio (Figure 6), a certain amount of unreacted reagents containing allyl groups and a small residue (5.8%) of vinyl groups remain in the system in addition to the product. The weight-average molecular weight (Mw) of the product was 30,800 Da. For the system where component 2p was used in excess (reaction C, Figure 6), virtually no reactants remained, indicating a complete reaction. The average molecular weight (Mw) of the product was 35,000 Da.

In addition to the experiments described above, we investigated the influence of the platinum catalyst on the selectivity of hydrosilylation toward different functional groups. For this purpose, reaction B was repeated under identical conditions, with the only difference being the platinum catalyst employed. Instead of Karstedt’s catalyst used previously, Speier’s catalyst was used. This experiment is designated as reaction Bs. Since reaction Bs was otherwise essentially identical to reaction B, the ^1^H NMR spectrum was recorded only after completion of the reaction.

**Figure 7:**
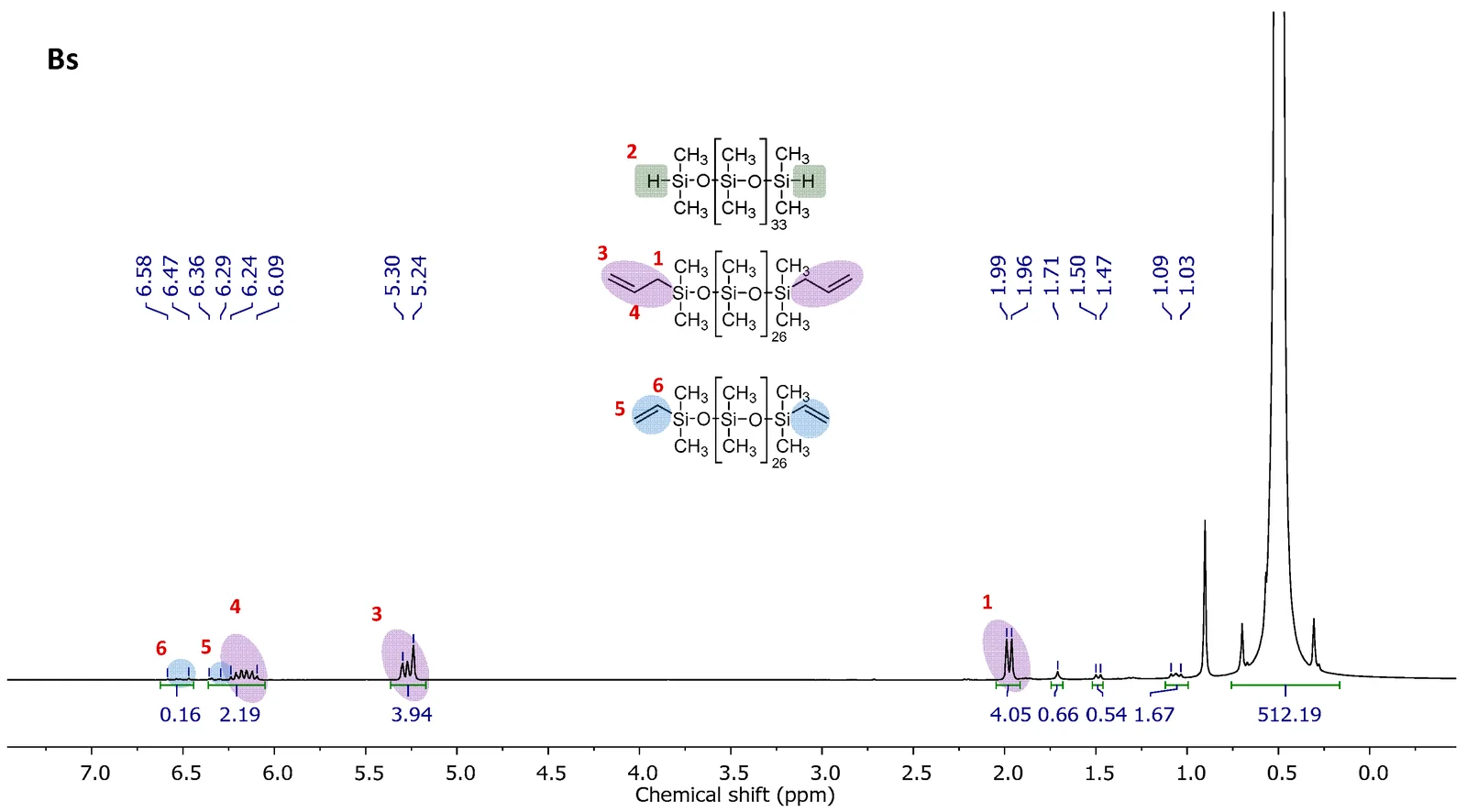
^1^H NMR spectrum of the reaction mixture after the reaction was complete (Bs). Colors denote the functional groups: purple—allyl group, blue—vinyl group, green—hydride.

The purpose of this experiment was not to investigate the rate of functional group consumption. However, a comparison of the final ^1^H NMR spectrum obtained for reaction Bs (Figure 7) with the corresponding spectrum for reaction B (Figure 4) indicates that replacement of the Pt^0^-based Karstedt’s catalyst with the Pt^IV^-based Speier’s catalyst has little effect on the selectivity of hydrosilylation toward allylsilyl and vinylsilyl groups.

**Scheme 4:**
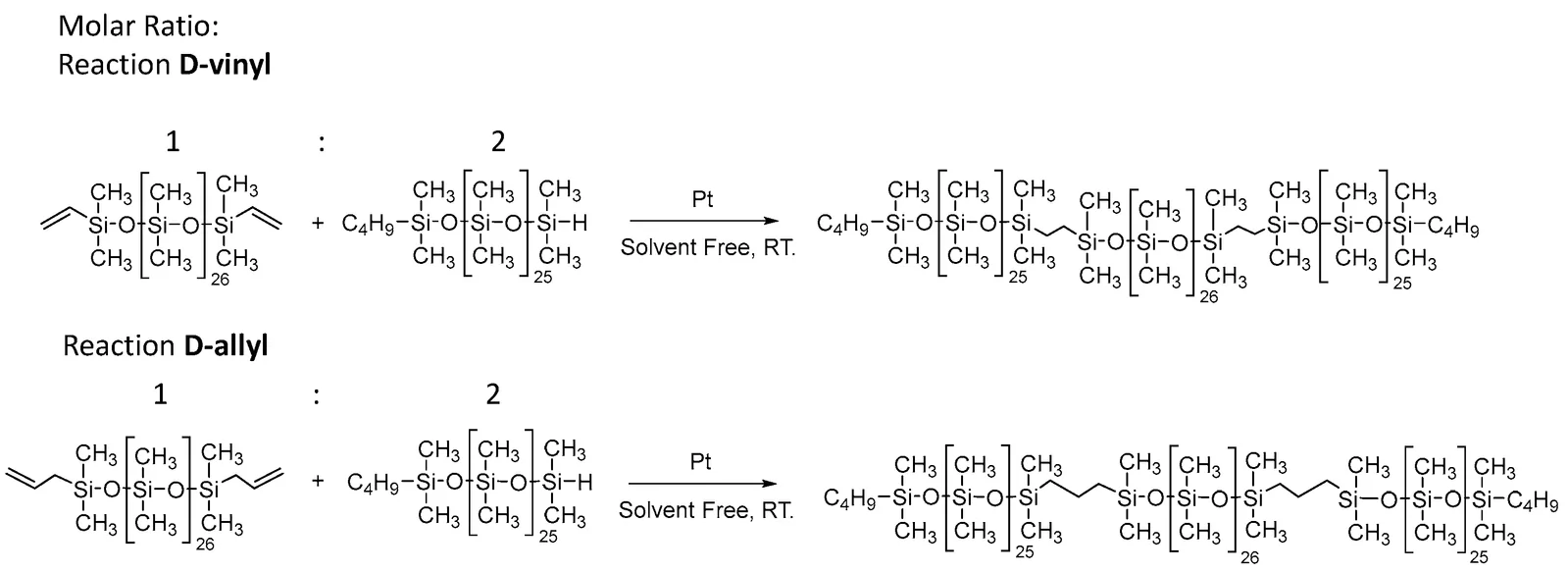
Model hydrosilylation reactions (reaction D-vinyl and reaction D-allyl). D-vinyl—hydrosilylation of 1 equiv of vinyl–Si (polymer 3p) with 2 equiv of H–Si (polymer 1p). D-allyl—hydrosilylation of 1 equiv of allyl–Si (polymer 4p) with 2 equiv of H–Si (polymer 1p).

To quantitatively compare the reactivity of allylsilyl and vinylsilyl groups, two parallel model hydrosilylation reactions were additionally performed, in which the consumption of functional groups was monitored as a function of time (Scheme 4). The first reaction was performed between compounds 1p and 3p (vinyl-terminated PDMS, reaction D-vinyl), whereas the second involved compounds 1p and 4p (allyl-terminated PDMS, reaction D-allyl). The reactants were mixed, the catalyst was added, and the reaction mixture was stirred for one minute before the mixture was transferred to an NMR tube. The reaction kinetics were subsequently monitored in the NMR tube without further stirring. The ^1^H NMR spectrum was recorded before catalyst addition, 5 min after catalyst addition, and at identical time intervals thereafter for both reactions. In all experiments, the platinum concentration was kept constant, and the reactants were used in equimolar amounts. The conversion of the functional groups was determined from the ^1^H NMR spectra (Figures S24 and S25, Supporting Information). It should be noted that a small amount of unreacted unsaturated groups remained even after completion of the reactions, despite the use of equimolar amounts of the starting reagents. This is attributable to uncertainties in determining the molecular weight of the polymers used as reactants by NMR spectroscopy, which caused the actual number of unsaturated groups in the reaction mixtures to slightly exceed the number of hydride groups within the limits of experimental error.

**Figure 8:**
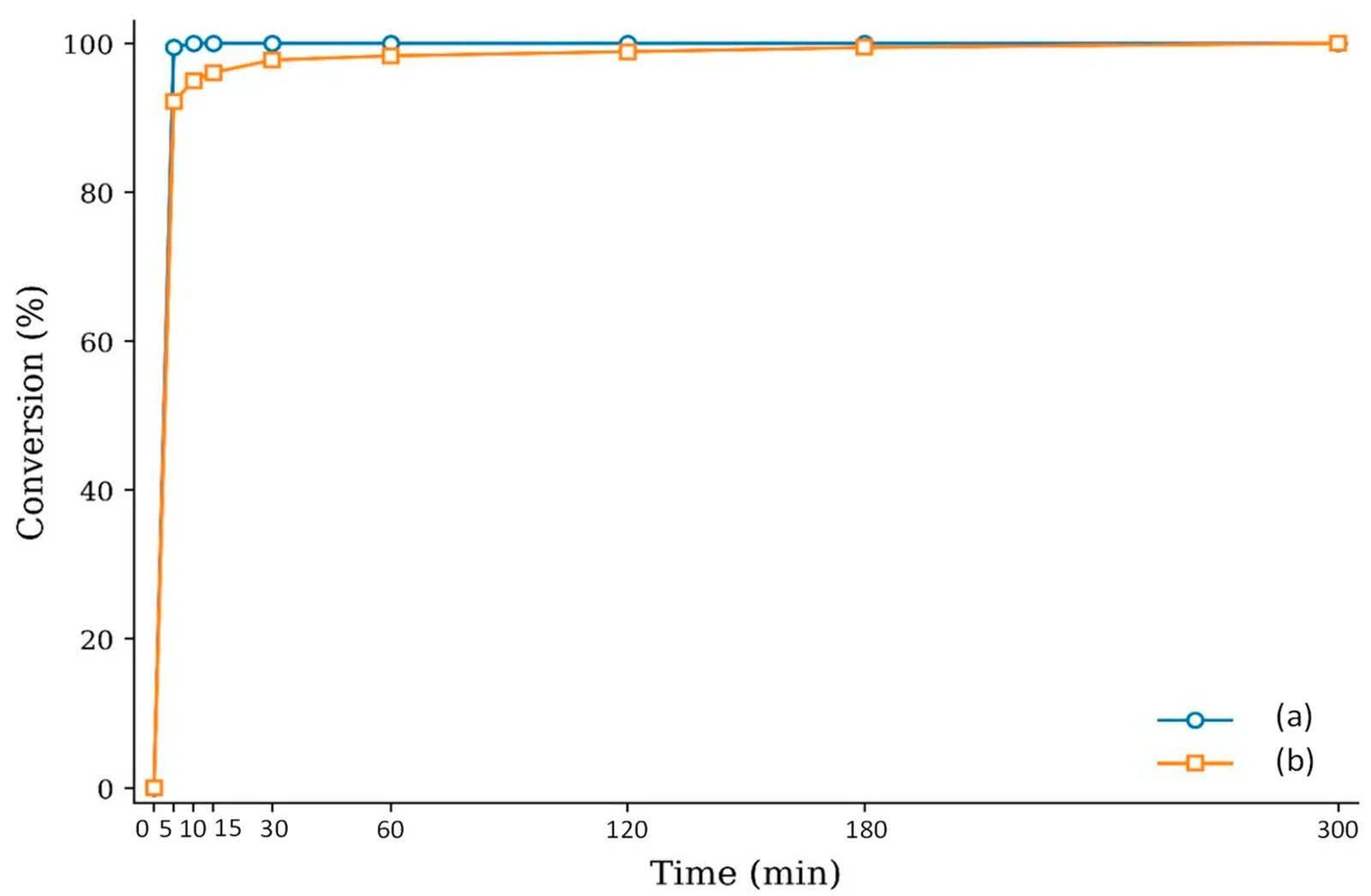
Experimental data for hydride functional groups vs. time. (a) Conversion of hydride functional groups in reaction D-vinyl. (b) Conversion of hydride functional groups in reaction D-allyl.

The resulting data were used to construct plots of Si–H conversion versus reaction time for reactions D-vinyl (Figure 8, curve a) and D-allyl (Figure 8, curve b).

The experimental results demonstrate that hydrosilylation proceeds rapidly in both systems. After 5 min, the Si–H conversion reached 99.46% for reaction D-vinyl and 92.18% for reaction D-allyl. In reaction D-vinyl, complete conversion was achieved after 10 min (Figure 8, curve a). In contrast, for reaction D-allyl, the conversion increased much more slowly thereafter, approaching completion only after approximately 5 h.

Analysis of the ^1^H NMR spectra of reaction D-allyl (Figure S25, Supporting Information) revealed the appearance of new signals at δ = 6.12–6.00 and 5.62–5.55 ppm, which were assigned to protons of isomerized allyl groups. This observation indicates that a competing allyl double-bond isomerization takes place during the hydrosilylation reaction. Similar side reactions have previously been reported for the hydrosilylation of allyl derivatives and other higher olefins [39]. In particular, Ref. [40] demonstrates that 1-octene begins to undergo isomerization to 2-octene immediately after the onset of hydrosilylation. The authors further reported that the rates of hydrosilylation and isomerization are comparable, although hydrosilylation remains the dominant process. The resulting 2-octene undergoes hydrosilylation much more slowly, ultimately yielding the same hydrosilylation product following reverse isomerization to 1-octene.

Based on these observations, we suggest that a similar process occurs in reaction D-allyl and reactions A, B and C. Although the terminal allylsilyl groups undergo rapid hydrosilylation at the initial stage of the reaction, their simultaneous isomerization may affect the rate of reaction. Accordingly, additional studies of the products of reaction D-allyl were performed using one-dimensional total correlation spectroscopy (TOCSY), ^13^C NMR, and ^1^H–^13^C HSQC NMR spectroscopy (Figures S27–S29, Supporting Information) to confirm the isomerization of the allyl groups.

Signs of allyl-group isomerization were also observed in reactions A, B, and C (Figure 2, Figure 4 and Figure 5). In reaction C, this is indicated by the proton signals in the region of δ = 6.20–6.09 ppm, marked as signal 9, which could be mistakenly assigned to the protons of the unreacted vinylsilyl groups remaining after the reaction; the proton signals in the region of δ = 5.69–5.63 ppm, marked as signal 8; and, most notably, the signal in the region of δ = 1.84–1.81 ppm, marked as signal 7, which can also be identified in reactions A and B.

**Figure 9:**
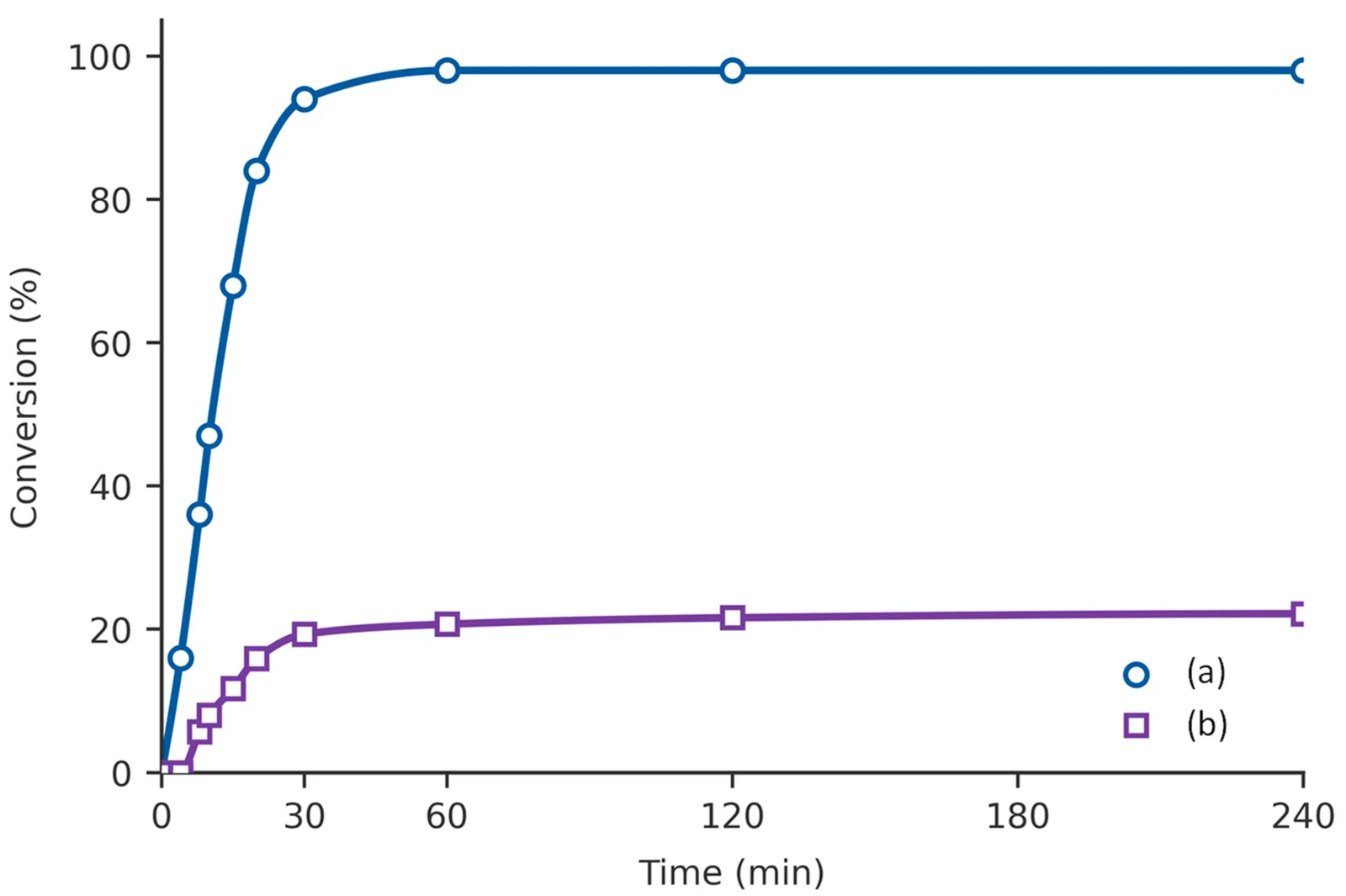
Conversion (%) of vinylsilyl and allylsilyl groups as a function of time (min). (a) Conversion of vinyl functional groups. (b) Conversion of allyl functional groups.

As an additional study, reaction A was repeated using a substantially lower platinum concentration in the reaction system—reaction E (2 ppm Pt). To reduce the platinum concentration, the commercially available Karstedt’s catalyst was diluted with ortho-xylene. The lower platinum concentration was chosen because the hydrosilylation reaction proceeds very rapidly, whereas the aim of this experiment was to investigate the processes occurring over time under competing reaction conditions. In addition, compound 5p, which has a higher molecular weight, was used instead of compound 4p in this experiment. We do not consider this difference to be critical for the assessment of relative reactivity, since the previous experiments had already demonstrated a substantial difference in the rates at which the functional groups undergo reaction. Reaction E differs from experiment D because, in reaction D, the compounds are not present under competing conditions and react individually at similar rates. Thus, based on the data obtained in reaction E (Table 3), the conversion of the unsaturated groups as a function of time was plotted (Figure 9).

The conversion of the vinylsilyl groups was determined from the change in the integral of the signal in the 6.14–6.03 ppm region. Unfortunately, quantitative determination of the allylsilyl group conversion is more challenging. We would like to emphasize that the ^1^H NMR spectra for reaction E were recorded without the addition of a deuterated solvent. The absence of a solvent affects the chemical shifts, resulting in overlap of the hydride group signal with the signal of the allyl group. In addition, the signal of the methylene protons of the allyl group may be affected by the water signal. Integration of the signals in the 0.7 to −0.5 ppm region is also difficult because the intense signal of methyl protons attached to silicon in the polymer distorts the baseline. Furthermore, the signal of the Si-CH_2_ protons partially overlaps with the signals of the methylene protons corresponding to the products of hydrosilylation of the allyl and vinyl groups (Figures S30 and S31, Supporting Information).

Therefore, the conversion of the allylsilyl groups was estimated based on the signal intensities in the 5.92–5.66 ppm region. However, the values obtained from this region show some inconsistencies. As an additional estimate, the signal assigned as signal 7 in Figure S31 was also considered. The data obtained from these signals were used to construct the dependence of allylsilyl group conversion on reaction time.

Thus, the experiment demonstrates that the allyl groups participate in the reaction simultaneously with the vinyl groups within the same reaction system, but at substantially different rates. Figures S30 and S31 in the Supporting Information present the spectra of the reaction mixture recorded before catalyst addition and at the indicated times after catalyst addition.

## 4. Conclusions

The results obtained demonstrate that, during the hydrosilylation reaction, the terminal vinyl groups in PDMS exhibit significantly greater reactivity than allyl groups. We hypothesize that the lower rate of hydrosilylation of the allylsilyl groups compared with the vinylsilyl groups observed under competing conditions in our system may be associated with the reversible isomerization of the allylic double bond. This process may contribute to the reduced rate of hydrosilylation of the allylsilyl groups. In contrast, vinylsilyl groups are not expected to undergo this competing process due to their molecular structure. However, despite the unambiguous results demonstrating that vinylsilyl groups undergo hydrosilylation faster than allylsilyl groups when both functional groups react simultaneously with hydride-terminated PDMS under the conditions studied, we cannot definitively determine the origin of this difference in reactivity based on the available data.

From a practical point of view, the established differences in reactivity can be considered a tool for the molecular and compositional design of silicone materials, allowing for a targeted influence on the process of structuring the polymer network and the properties of the final material.

The obtained data highlight the importance of fine-tuning the chemical activity of functional fragments in the creation of silicone compositions with specified performance properties and demonstrate the potential of using differences in reactivity as one of the key parameters of molecular design.

In future studies, the approach presented in this work can be extended through a systematic investigation of the effects of temperature and catalyst concentration on the relative reactivity of the functional groups. Additionally, it is of interest to further expand the proposed approach to systems with different functional unit architectures, such as PDMS with side-mounted unsaturated groups, as well as polyorganosiloxanes with silsesquioxane fragments. Research on specially synthesized model compounds will allow us to determine the influence of the molecular environment and spatial organization of functional groups on their relative reactivity in the hydrosilylation reaction.

## Figures and Tables

**Table 1:** Molecular weight characteristics of oligomers.

№	Functional Group	F ^1^	Mn, Da	Mw, Da	Mw/Mn	M_NMR_
1p	Hydride	1	2270	2700	1.2	2000
2p	Hydride	2	2670	3920	1.5	2630
3p	Vinyl	2	2120	3700	1.7	2050
4p	Allyl	2	2140	3540	1.7	2100
5p	Allyl	2	3050	5450	1.8	2530

^1^ The number of functional groups per oligomer molecule.

**Table 2:** Theoretical and NMR-derived functional group ratio in the actual reaction mixture.

Reaction	Functional Group Ratio						Conversion, %		
	Vi	All	Si-H	Vi	All	Si-H	Vi	All	Si-H
	Theoretical			NMR					
A	1	1	1	1	1.13	1.06	96.6	10.5	100
B	1	1	1	1	1.16	0.99	94.2	11.3	100
C	1	1	1.5	1	1.16	1.52	100	56	100
D-Vi	1	-	1	1	-	0.93	91.5	-	100
D-All	-	1	1	-	1	1	-	92	100
E	1	1	1	1	0.91	1.24	100	25.5	100

**Table 3:** Conversion of vinylsilyl and allylsilyl groups as a function of time.

Time (min)	Conversion of Vinyl (%)	Conversion of Allyl (%) (Based on 5.92–5.66 pmm)	Conversion of Allyl (%) (Based on Area № 7)
0	0	0	0
4	16	0	0
8	36	8	5.7
10	47	7	8
15	68	8	11.7
20	84	15	15.9
30	94	19	19.3
60	98	18	20.7
120	98	19	21.6
240	98	16	22.16
2 days	100	25	25

## Data Availability

The original contributions presented in this study are included in the article/Supplementary Material. Further inquiries can be directed to the corresponding author.

## Supplementary Materials

The following supporting information can be downloaded at https://www.mdpi.com/article/10.3390/polym18192373/s1, Figure S1: ^1^H NMR spectrum of α-(butyldimethyl)-ꞷ-(dimethyl)-polydimethylsiloxane; Figure S2: ^1^H NMR spectrum of α,ꞷ-(dimethyl)-polydimethylsiloxane; Figure S3: ^1^H NMR spectrum of α,ꞷ-(allyldimethyl)-polydimethylsiloxane; Figure S4: ^1^H NMR spectrum of α,ꞷ-(vinyldimethyl)-polydimethylsiloxane; Figure S5: GPC curve of α-(butyldimethyl)-ꞷ-(dimethyl)-polydimethylsiloxane; Figure S6: GPC curve of α,ꞷ-(dimethyl)-polydimethylsiloxane; Figure S7: GPC curve of α,ꞷ-(vinyldimethyl)-polydimethylsiloxane; Figure S8: GPC curve of α,ꞷ-(allyldimethyl)-polydimethylsiloxane; Figure S9: ^1^H NMR spectrum of reaction mixture A before adding Karstedt’s catalyst; Figure S10: ^1^H NMR spectrum of reaction mixture A after reaction; Figure S11: ^29^Si NMR spectrum of reaction mixture A before adding Karstedt’s catalyst with an expanded view of the 10 to −25 ppm region; Figure S12: ^29^Si NMR spectrum of the reaction mixture A after reaction with an expanded view of the 10 to −25 ppm region; Figure S13: ^1^H NMR spectrum of reaction mixture B before adding Karstedt’s catalyst; Figure S14: ^1^H NMR spectrum of reaction mixture B after reaction; Figure S15: ^29^Si NMR spectrum of reaction mixture B before adding Karstedt’s catalyst with an expanded view of the 10 to −25 ppm region; Figure S16: ^29^Si NMR spectrum of reaction mixture B after reaction with an expanded view of the 10 to −25 ppm region; Figure S17: {^1^H, ^29^Si} HMBC spectrum of mixture B after reaction; Figure S18: ^1^H NMR spectrum of reaction mixture C before adding Karstedt’s catalyst; Figure S19: ^1^H NMR spectrum of reaction mixture C after reaction; Figure S20: ^29^Si NMR spectrum of reaction mixture C before adding Karstedt’s catalyst with an expanded view of the 10 to −25 ppm region; Figure S21: ^29^Si NMR spectrum of reaction mixture C after reaction with an expanded view of the 10 to −25 ppm region; Figure S22: ^1^H NMR spectrum of reaction mixture Bs after reaction; Figure S23: ^29^Si NMR spectrum of reaction mixture Bs after reaction with an expanded view of the 10 to −25 ppm region; Figure S24: ^1^H NMR spectra of reaction mixture D-vinyl; Figure S25: 1H NMR spectra of reaction mixture D-allyl; Figure S26: Experimental data for hydride functional groups vs. time. (a) Conversion of hydride functional groups in reaction D-vinyl. (b) Conversion of hydride functional groups in reaction D-allyl; Figure S27: ^1^H NMR spectra of the reaction mixture of reaction D-allyl before adding catalyst, after the reaction and one-dimensional total correlation spectroscopy; Figure S28: ^1^H—^13^C HSQC NMR spectrum of the reaction mixture of reaction D-allyl; Figure S29: ^13^C NMR spectrum of the reaction mixture of reaction D-allyl with an expanded view of the 150 to 105 ppm region; Figure S30: ^1^H NMR spectra of reaction mixture E before adding catalyst and over a period of 2 days after catalyst addition (a) and with an expanded view of the 6.5 to 4.3 ppm region (b); Figure S31: ^1^H NMR spectra of reaction mixture E with a view of the 1.6 to −0.3 ppm region; Figure S32: Conversion (%) of vinylsilyl and allylsilyl groups as a function of time (min); Figure S33: GPC curve of reaction mixture A before adding Karstedt’s catalyst; Figure S34: GPC curve of reaction mixture A after reaction; Figure S35: GPC curve of reaction mixture B before adding Karstedt’s catalyst; Figure S36: GPC curve of reaction mixture B after reaction; Figure S37: GPC curve of reaction mixture C before adding Karstedt’s catalyst; Figure S38: GPC curve of reaction mixture C after reaction.

## Abbreviations

The following abbreviations are used in this manuscript:

D_3_	Hexamethylcyclotrisiloxane
D_4_	Octamethylcyclotetrasiloxane
GPC	Gel permeation chromatography
NMR	Nuclear magnetic resonance
PDMS	Polydimethylsiloxane oligomers
THF	Tetrahydrofuran
TMAH	Tetramethylammonium hydroxide
TMDS	1,1,3,3-tetramethyldisiloxane
