# Supplementary material for: Comparison of the Reactivity of PDMS with Terminal Allyl and Vinyl Groups in the Hydrosilylation Reaction
Source: Polymers (Basel). 2026 Sep 29;18(19):2373. doi: 10.3390/polym18192373 (PMC13666875; doi:10.3390/polym18192373)
Supplement: Supplementary file 1 [file polymers-18-02373-s001.zip › polymers-4541101-supplementary.pdf]

Supplementary data

Comparison of the reactivity of PDMS with terminal allyl and vinyl groups in the hydrosilylation reaction

Valentin Gubarev[1], Fedor Krylov[1], Sofia Ardabevskaia[1,2], Kseniya Bezlepkina[1,2], Kirill Bakanov[1,2], Sergey Milenin[1,2]*, and Aziz Muzafarov[1]

[1] Enikolopov Institute of Synthetic Polymeric Materials, Russian Academy of Sciences, Profsoyuznaya 70, 117393 Moscow, Russia
[2] Research Laboratory of New Silicone Materials and Technologies, Tula State Lev Tolstoy Pedagogical University, 300026 Tula, Russia
* Correspondence: cephe@mail.ru

^{1}H, ^{13}C, and ^{29}Si NMR spectra and GPC curves of the initial reagents and reaction products.

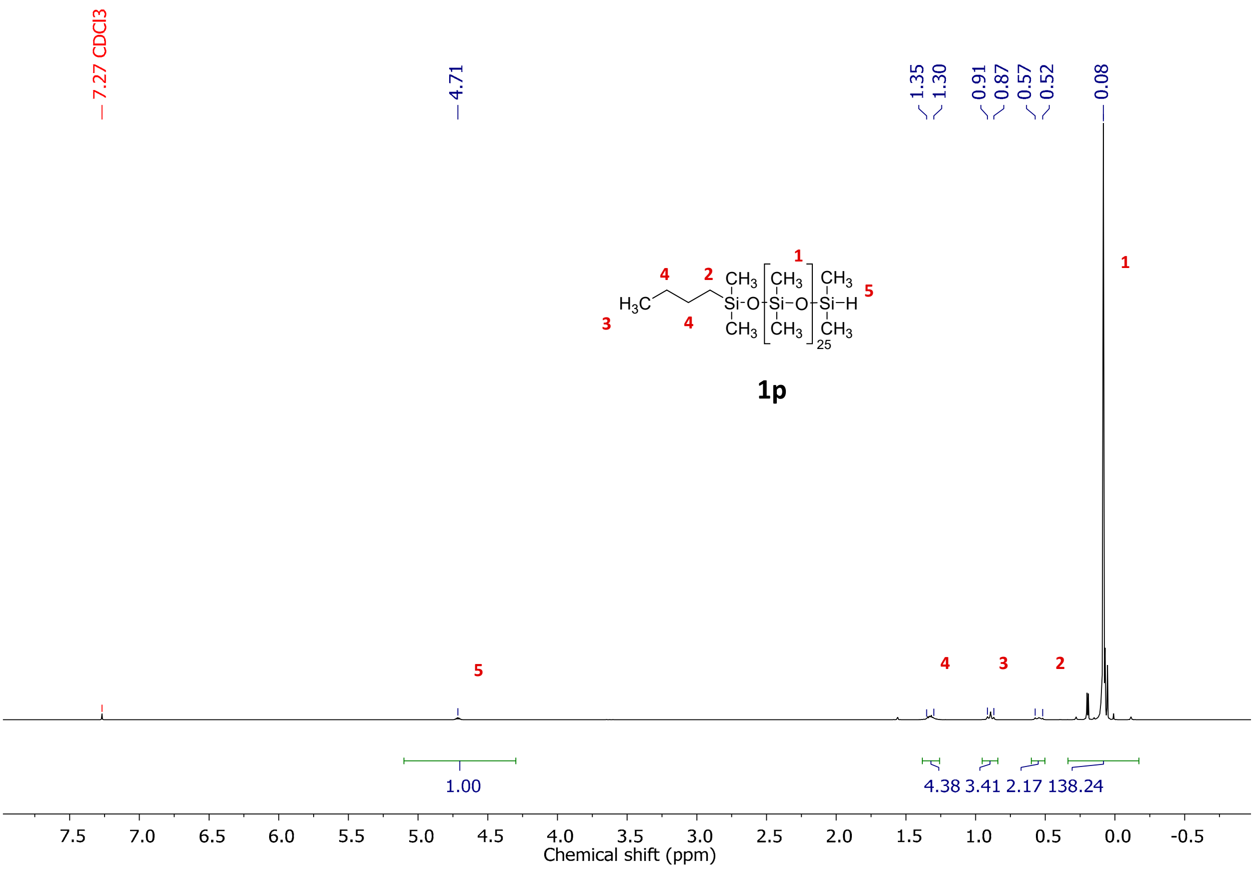

Figure S1. ^{1}H NMR spectrum of α-(butyldimethyl)-ω-(dimethyl)-polydimethylsiloxane.

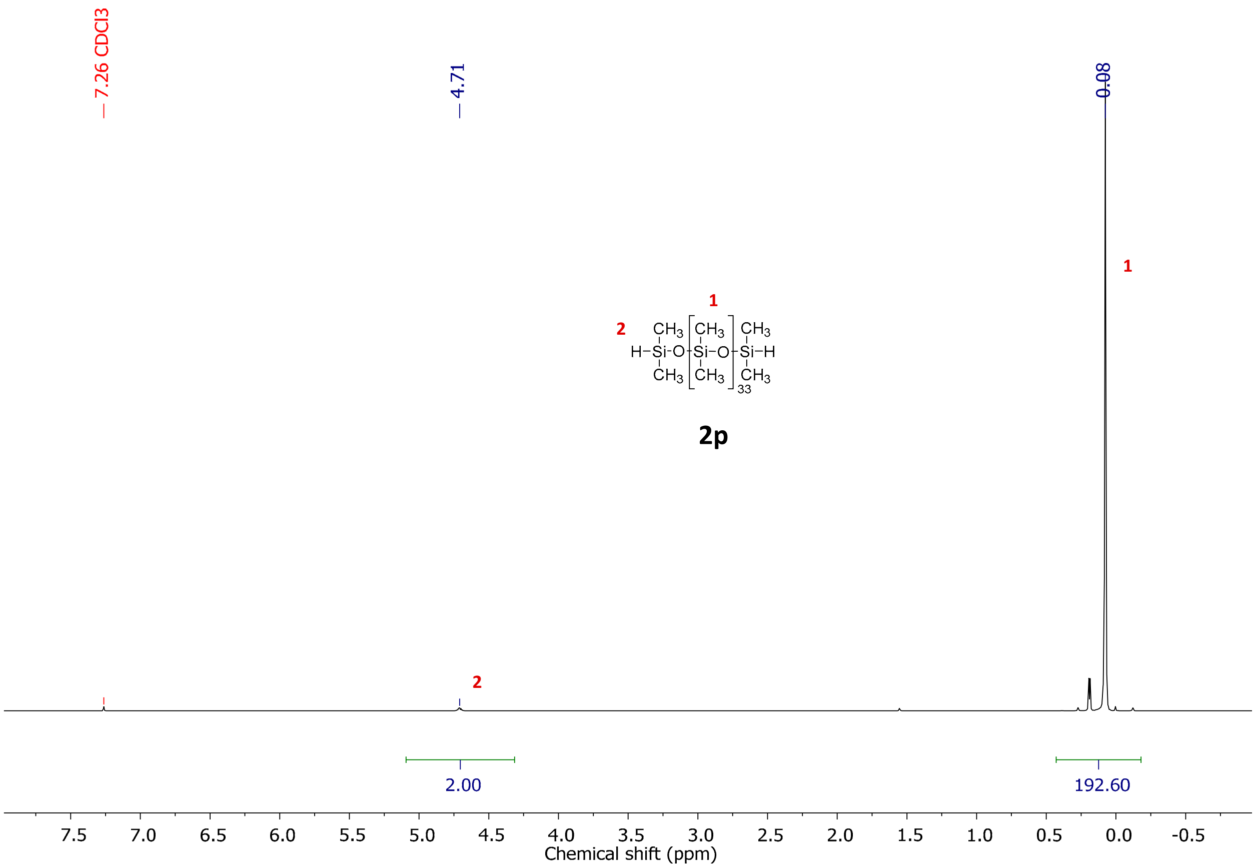

Figure S2. ^{1}H NMR spectrum of α,ω-(dimethyl)-polydimethylsiloxane.

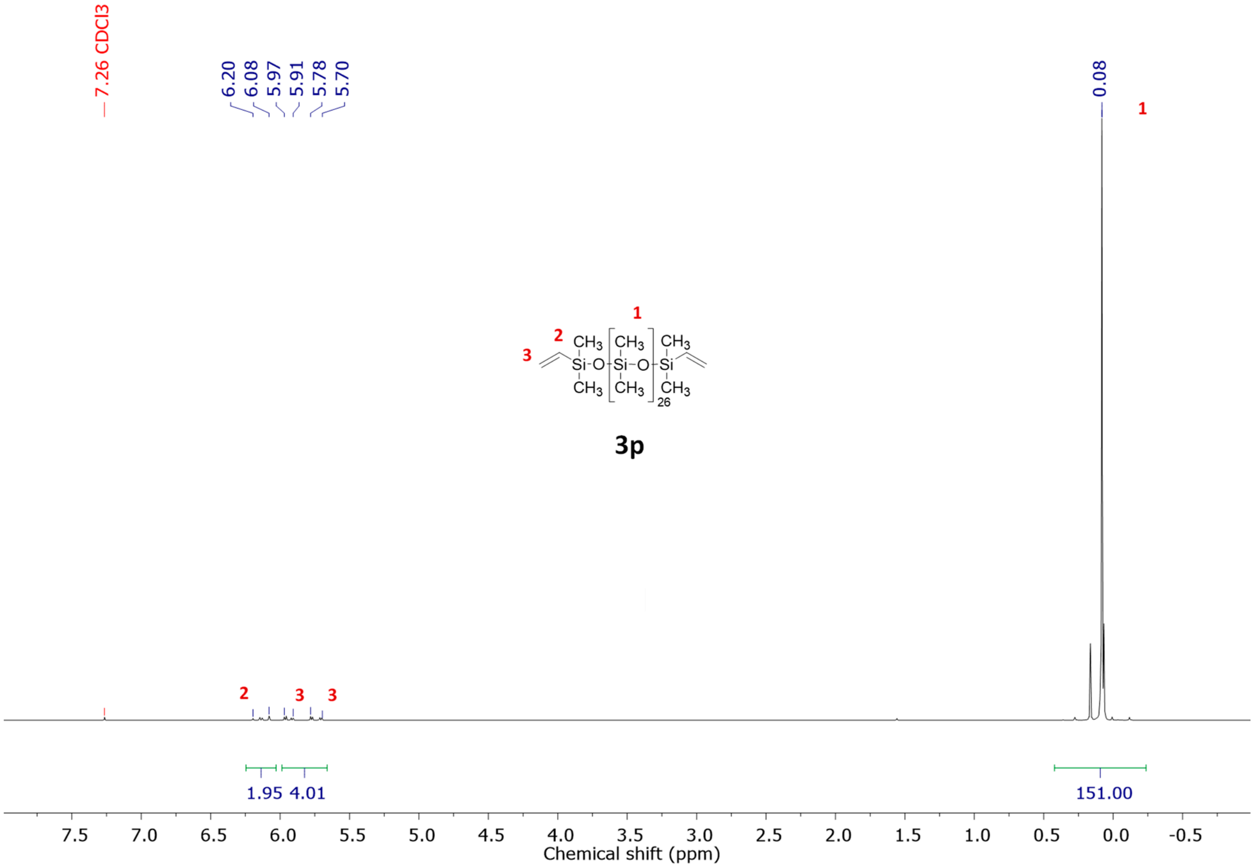

Figure S3. ^{1}H NMR spectrum of α,ω-(allyldimethyl)-polydimethylsiloxane.

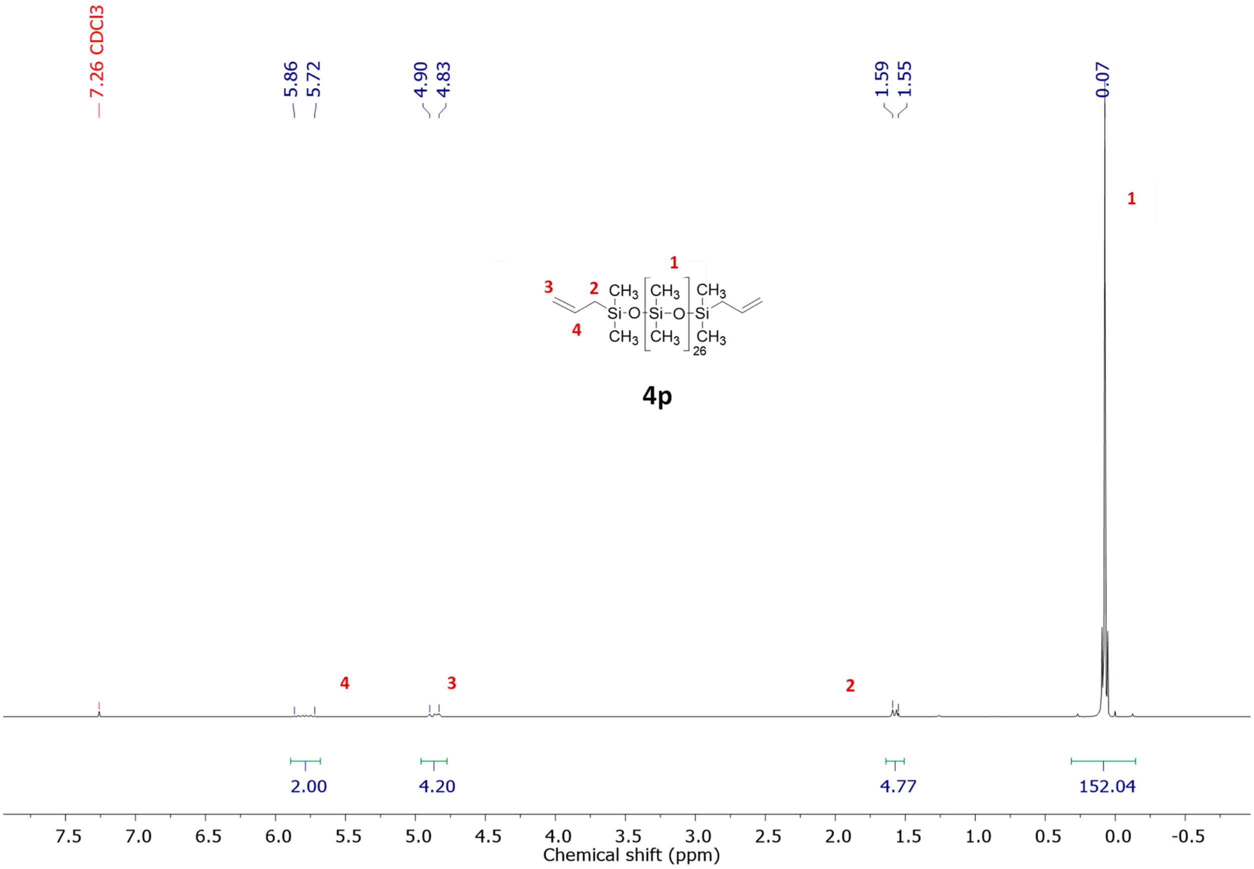

Figure S4. ^{1}H NMR spectrum of α,ω-(vinyldimethyl)-polydimethylsiloxane.

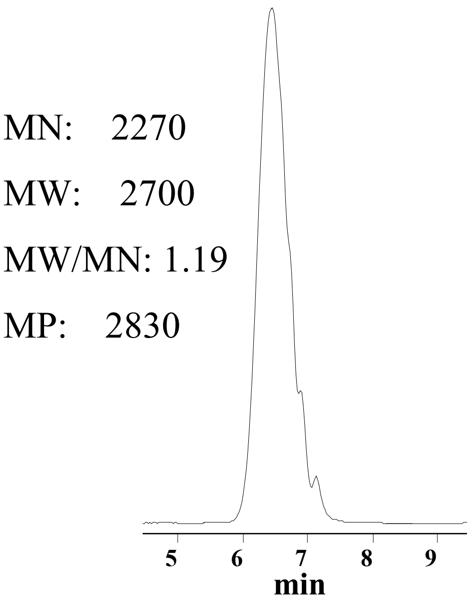

Figure S5. GPC curve of α-(butyldimethyl)-ω-(dimethyl)-polydimethylsiloxane.

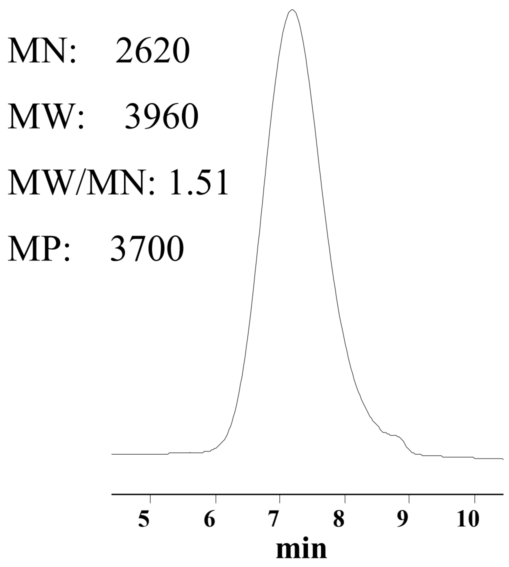

Figure S6. GPC curve of α,ω-(dimethyl)-polydimethylsiloxane.

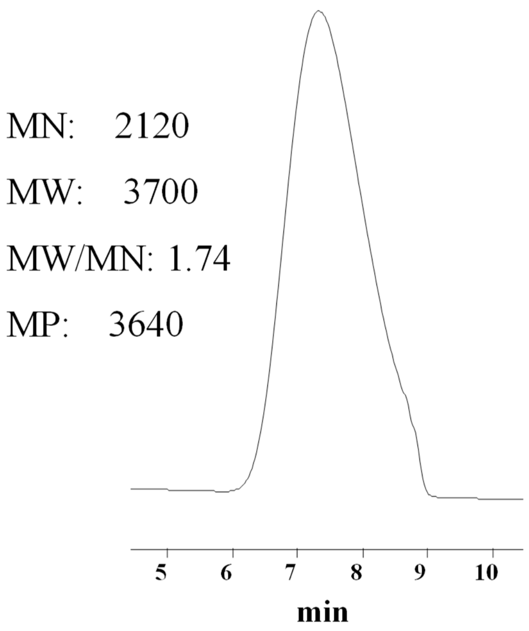

Figure S7. GPC curve of α,ω-(vinyldimethyl)-polydimethylsiloxane.

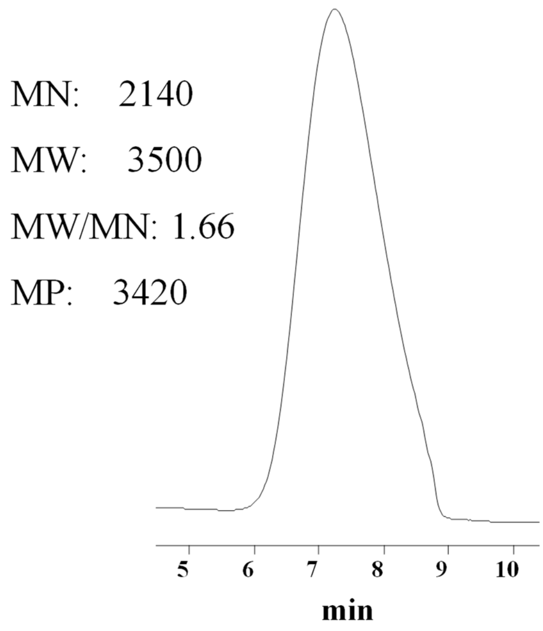

Figure S8. GPC curve of α,ω-(allyldimethyl)-polydimethylsiloxane.

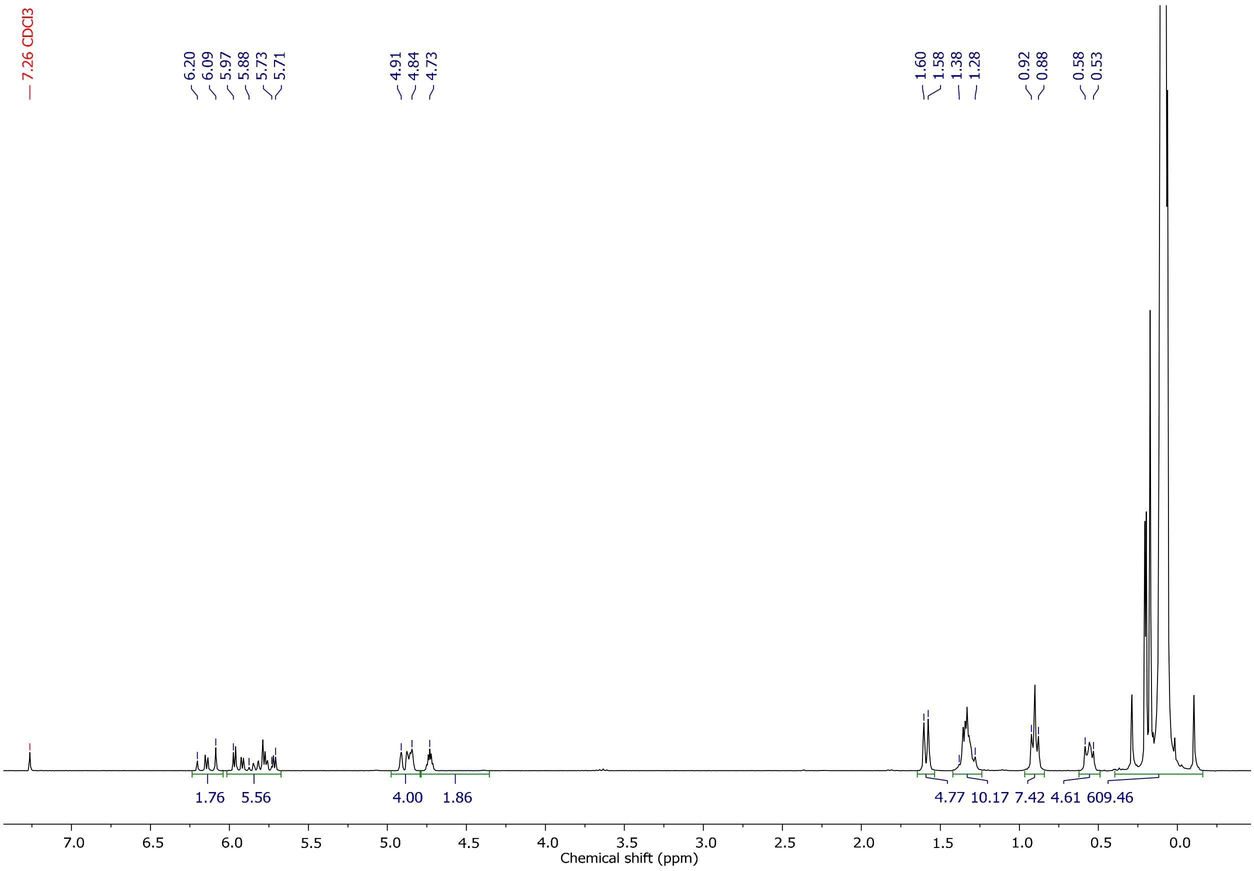

Figure S9. ^{1}H NMR spectrum of reaction mixture A before adding Karstedt’s catalyst.

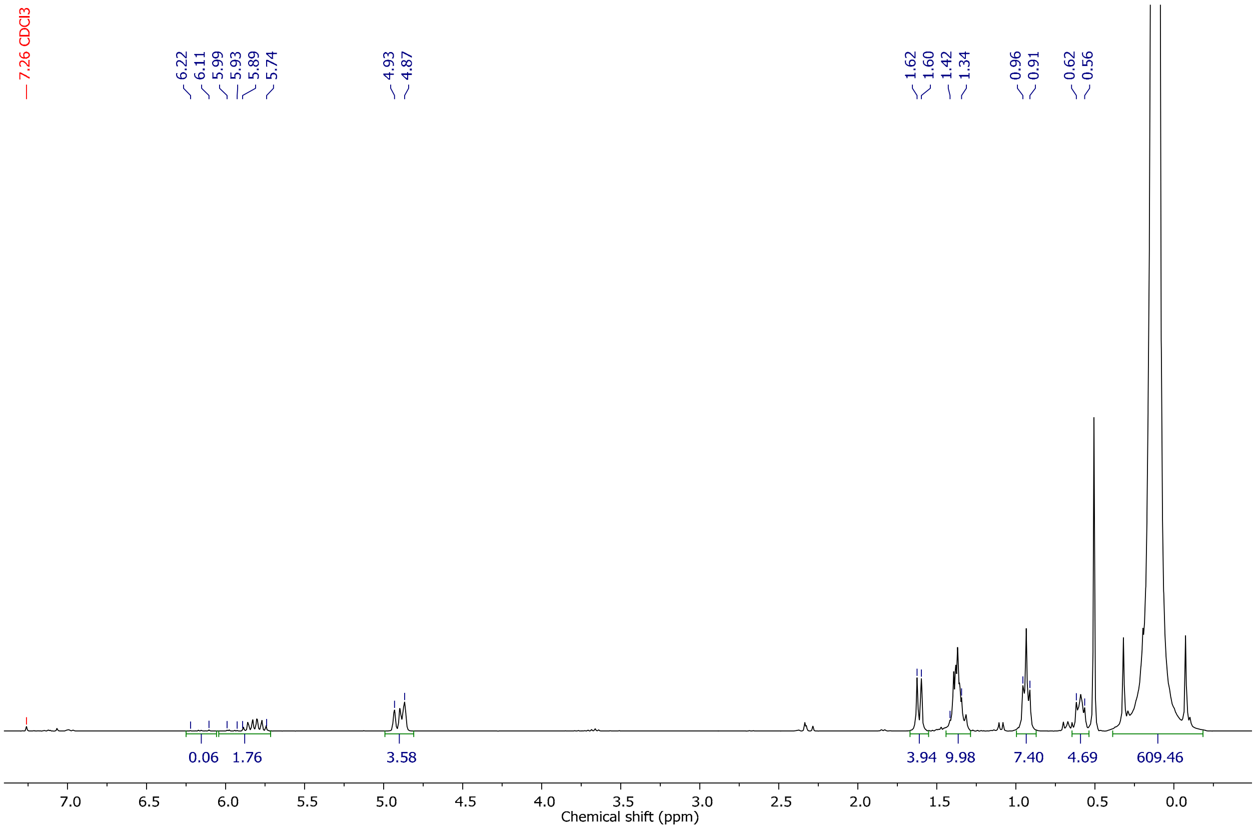

Figure S10. ^1H NMR spectrum of reaction mixture A after reaction.

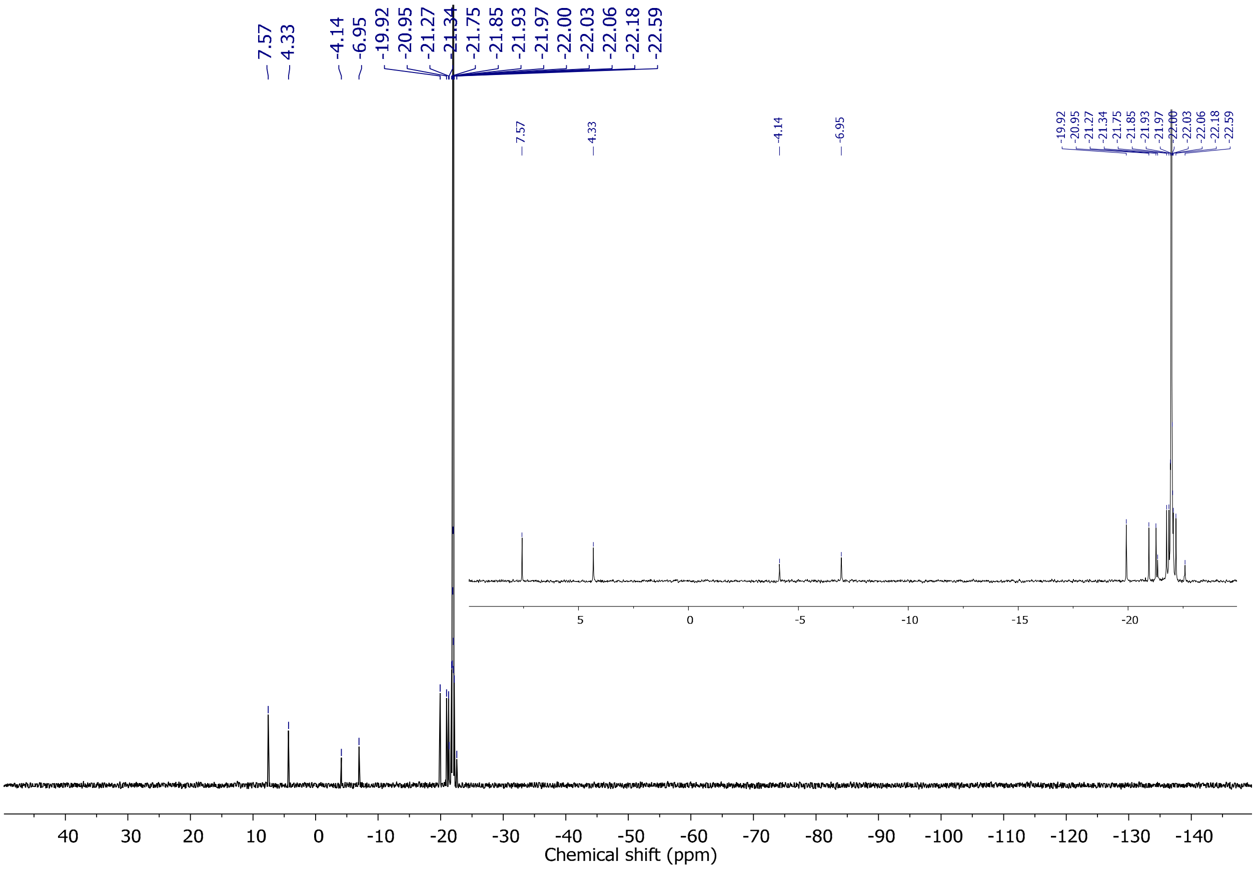

Figure S11. ^{29}Si NMR spectrum of reaction mixture A before adding Karstedt's catalyst with an expanded view of the 10 to -25 ppm region.

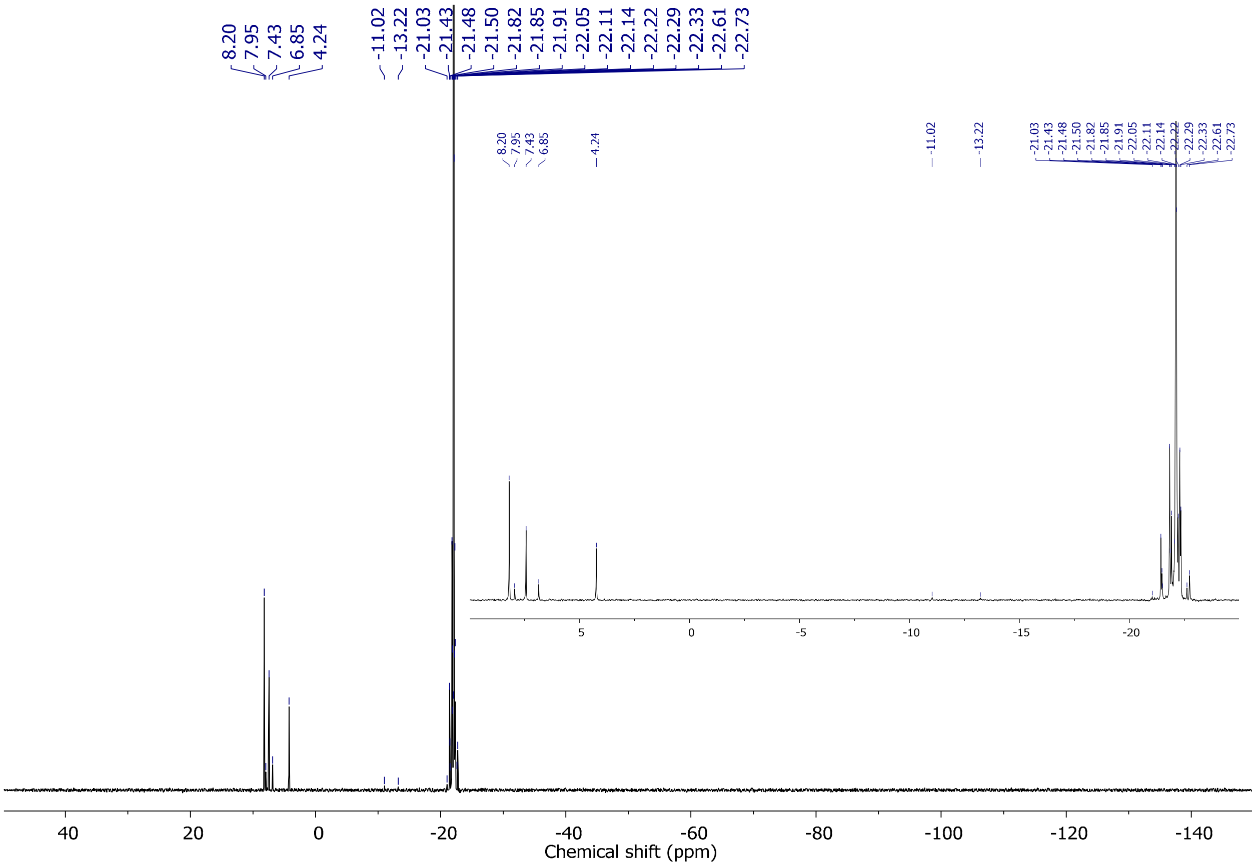

Figure S12. ^{29}Si NMR spectrum of reaction mixture A after reaction with an expanded view of the 10 to -25 ppm region.

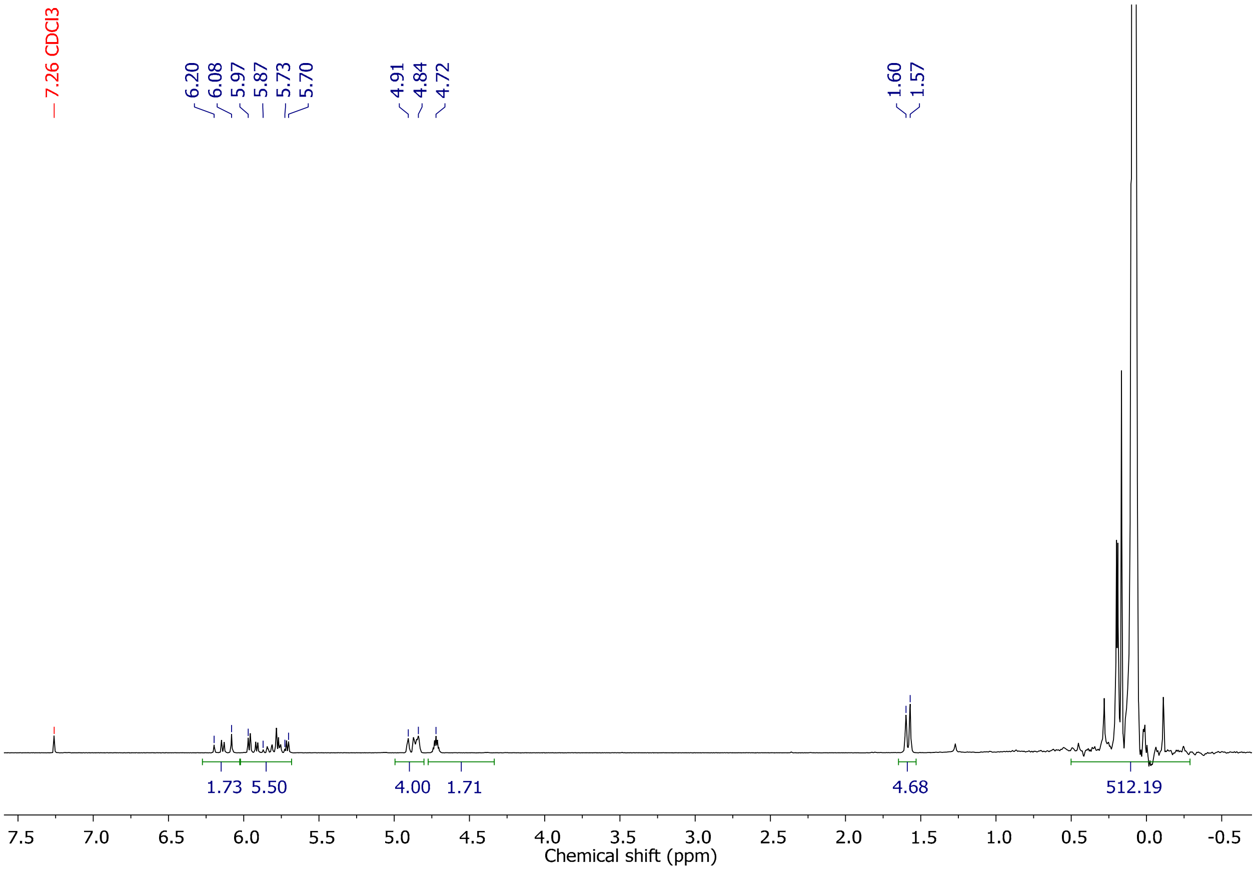

Figure S13. ^{1}H NMR spectrum of reaction mixture B before adding Karstedt's catalyst.

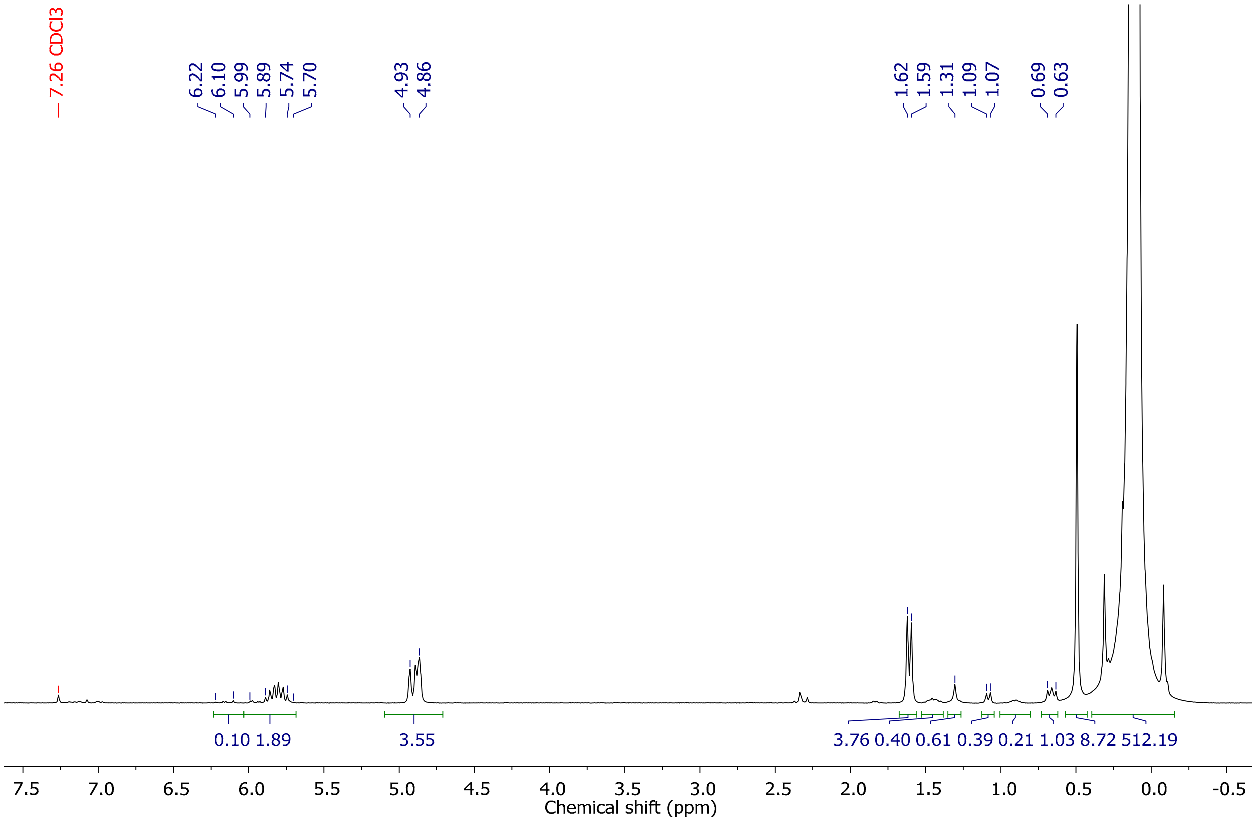

Figure S14. ^{1}H NMR spectrum of reaction mixture B after reaction.

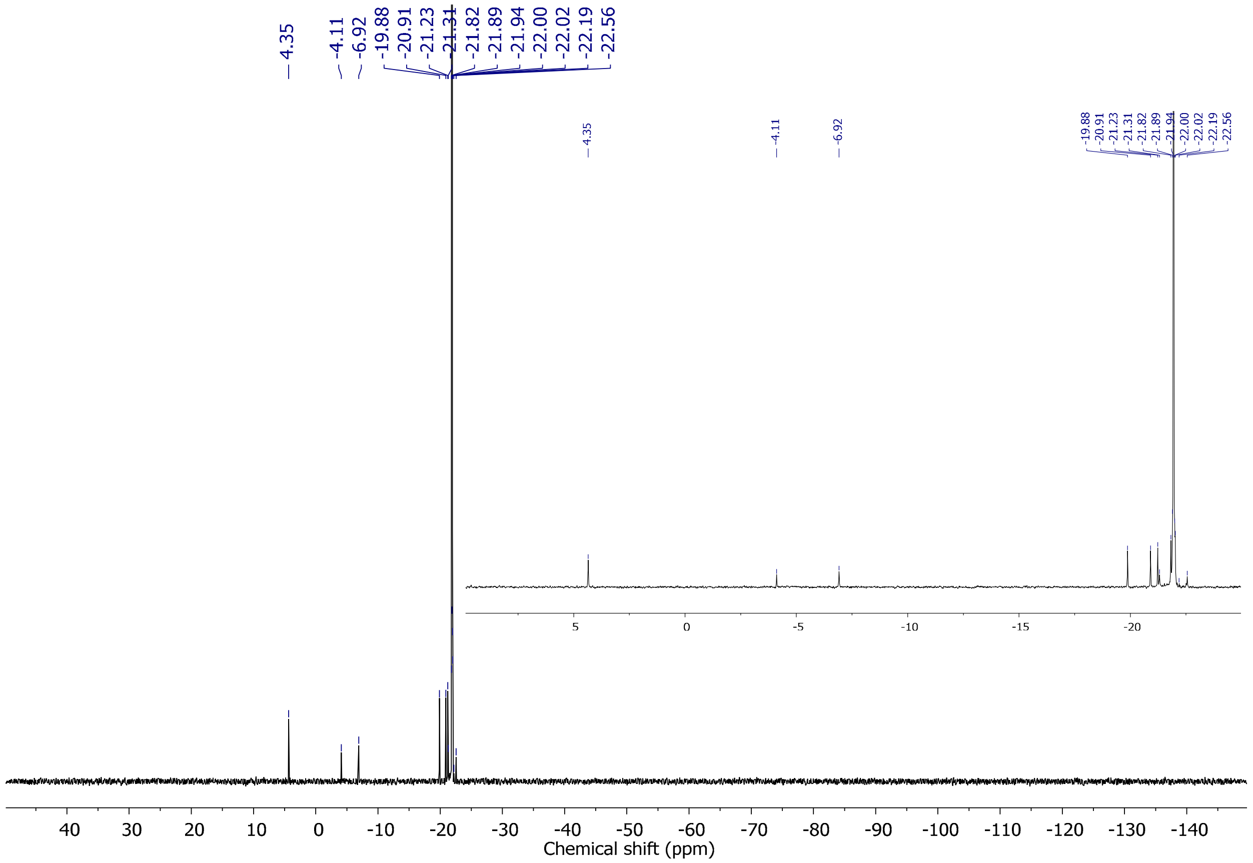

Figure S15. ^{29}Si NMR spectrum of reaction mixture B before adding Karstedt's catalyst with an expanded view of the 10 to -25 ppm region.

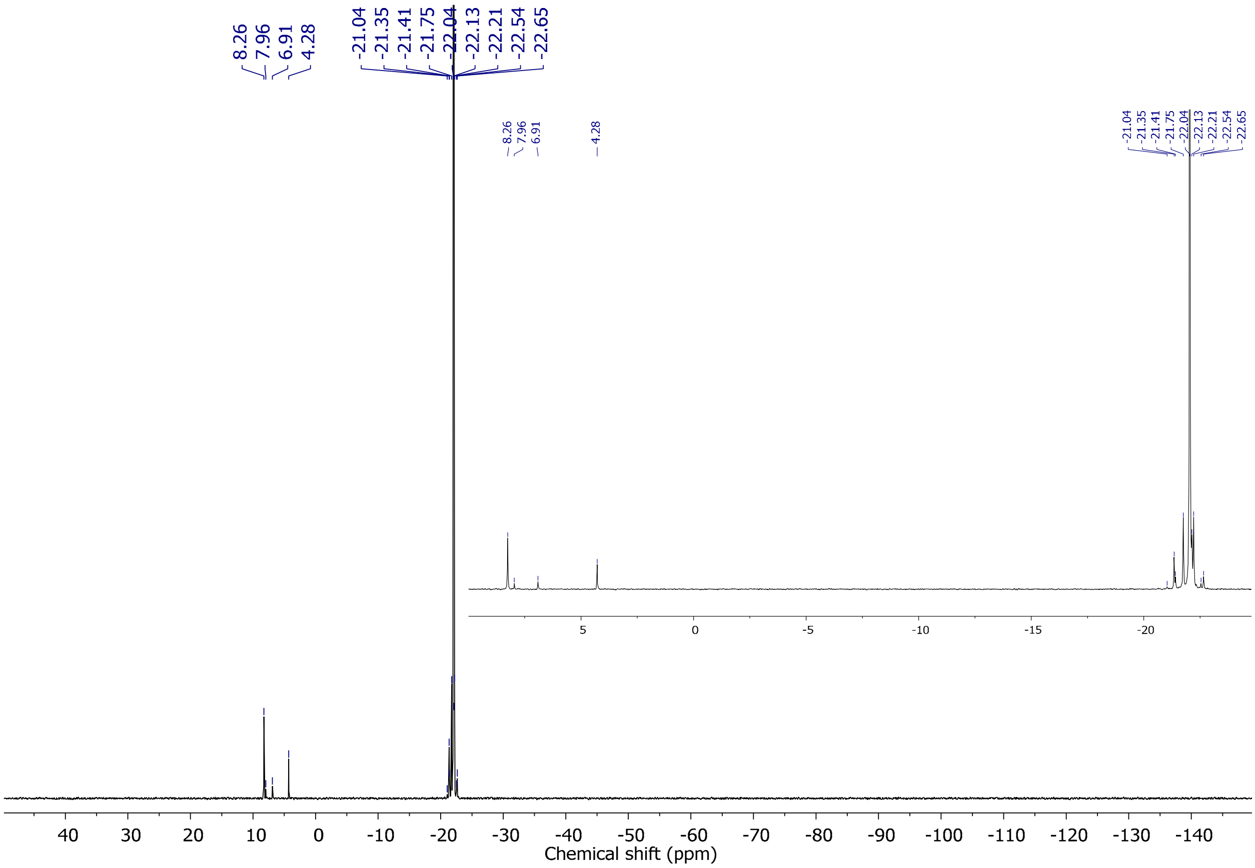

Figure S16. ^{29}Si NMR spectrum of reaction mixture B after reaction with an expanded view of the 10 to -25 ppm region.

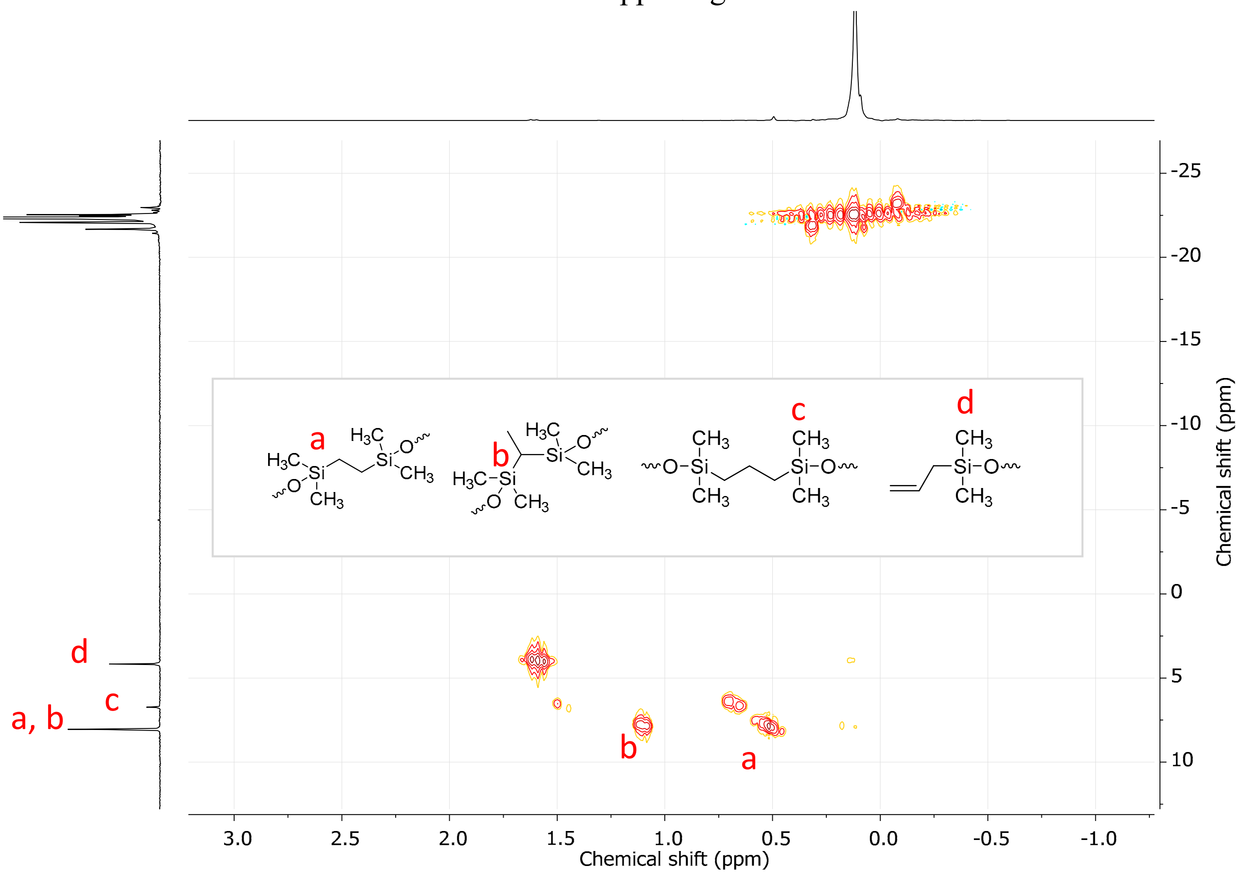

Figure S17. {^{1}H, ^{29}Si} HMBC spectrum of mixture B after reaction.

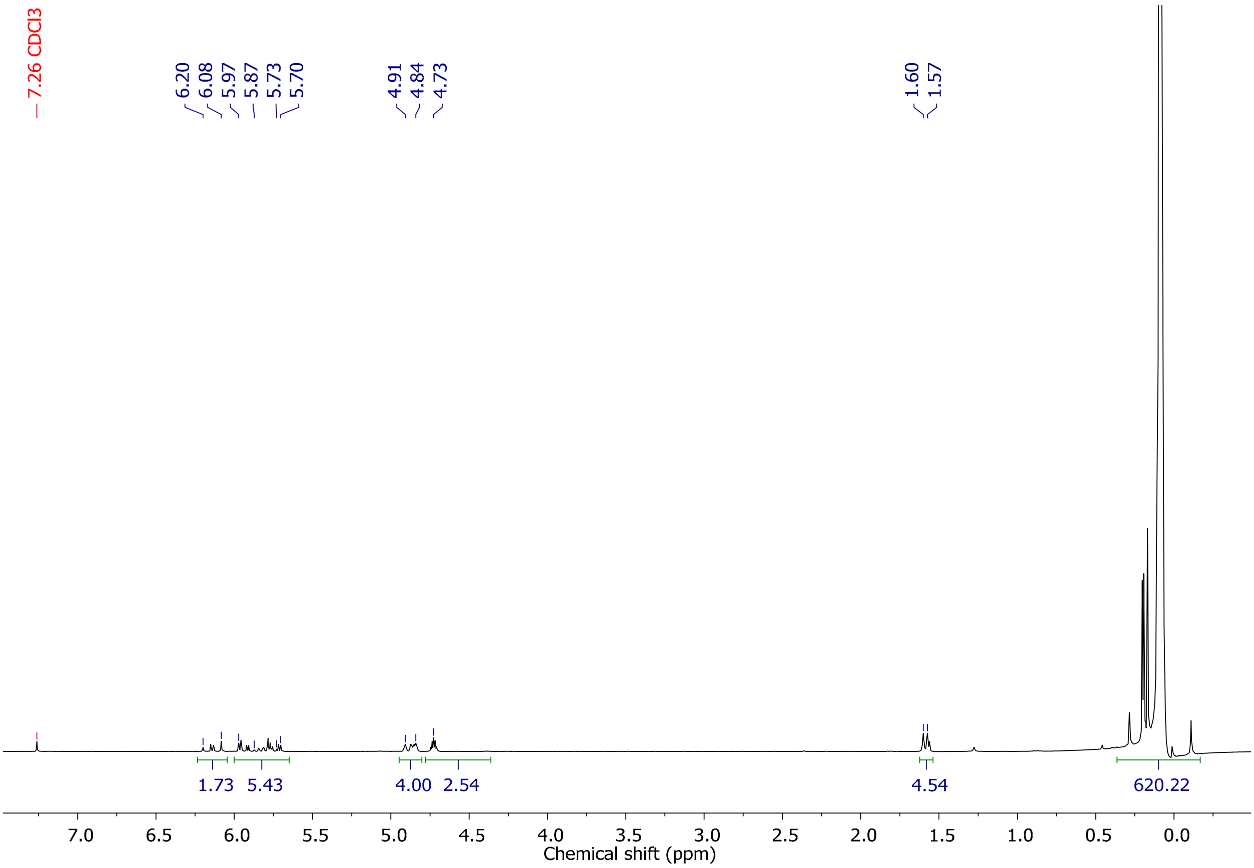

Figure S18. ^{1}H NMR spectrum of reaction mixture C before adding Karstedt's catalyst.

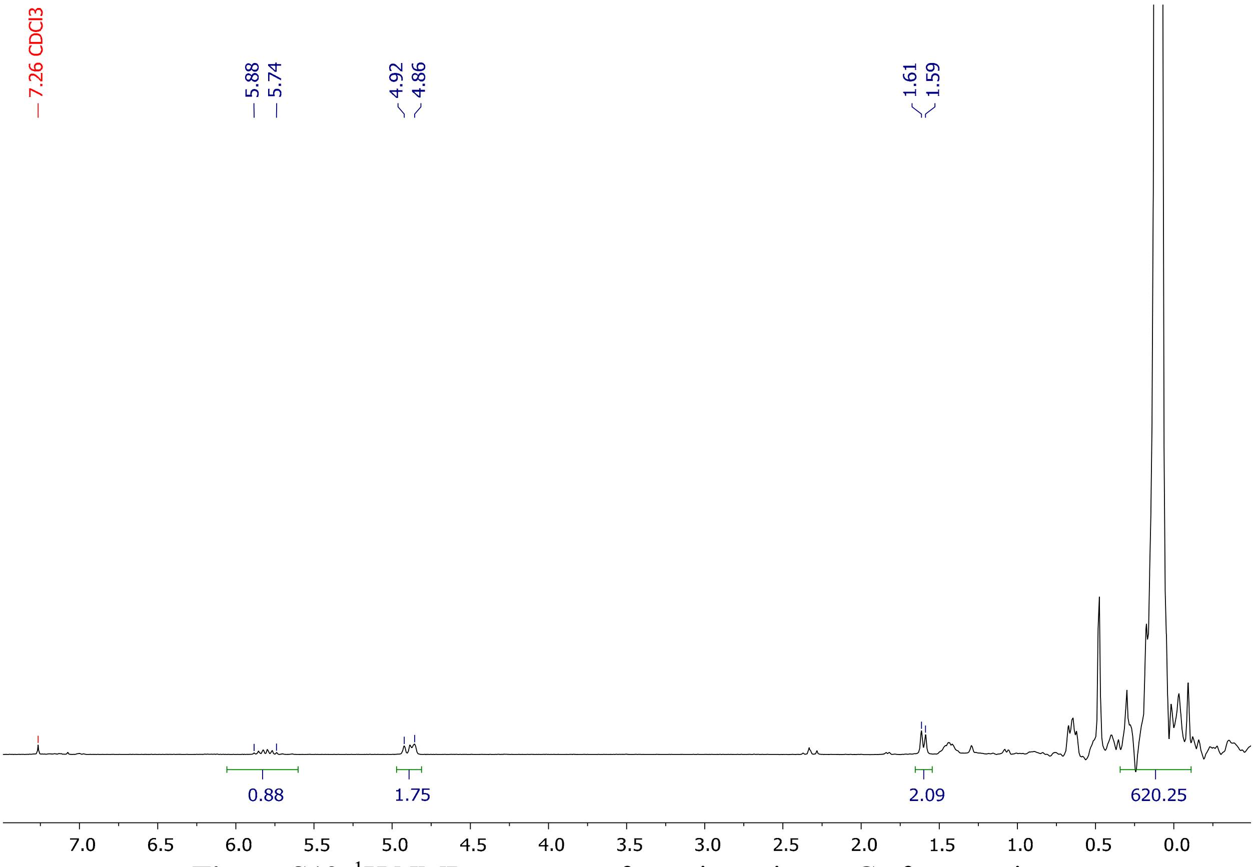

Figure S19. ^{1}H NMR spectrum of reaction mixture C after reaction.

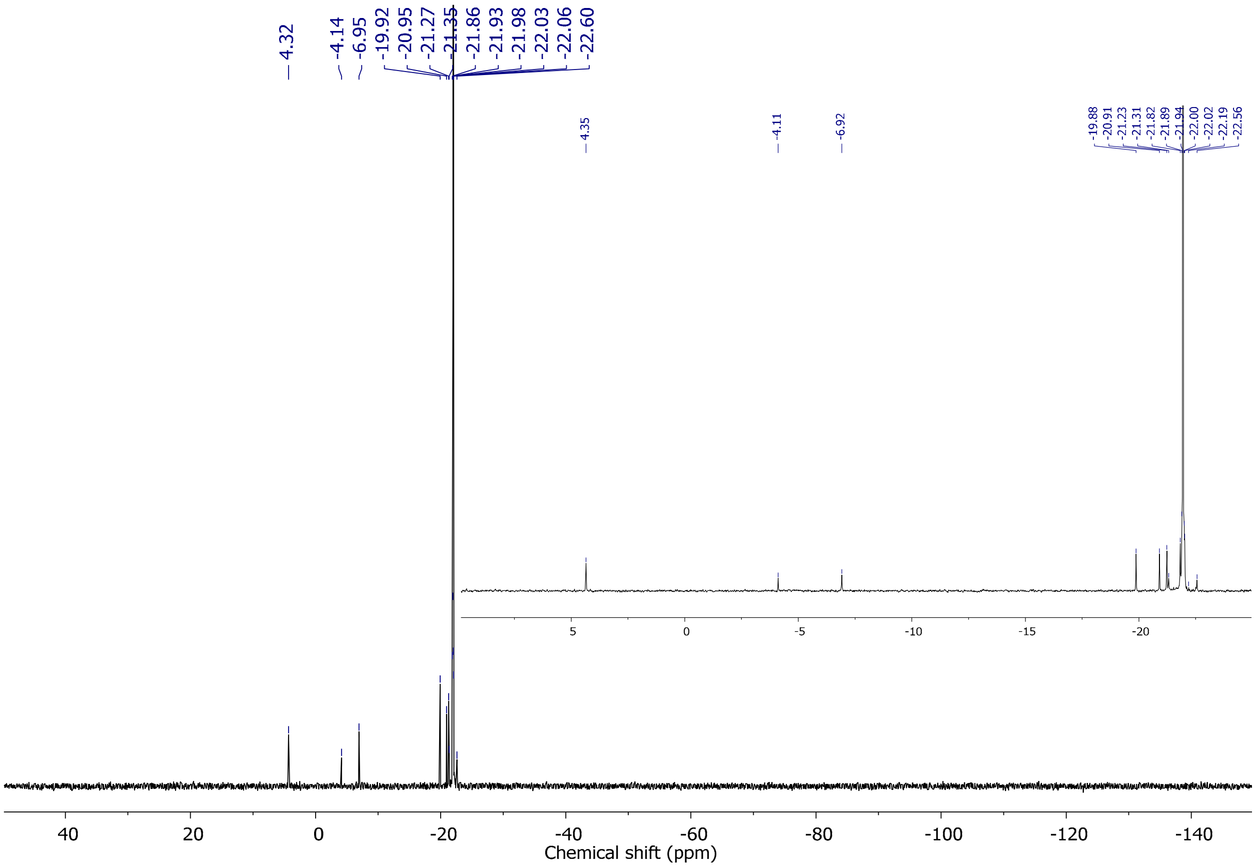

Figure S20. ^{29}Si NMR spectrum of reaction mixture C before adding Karstedt's catalyst with an expanded view of the 10 to -25 ppm region.

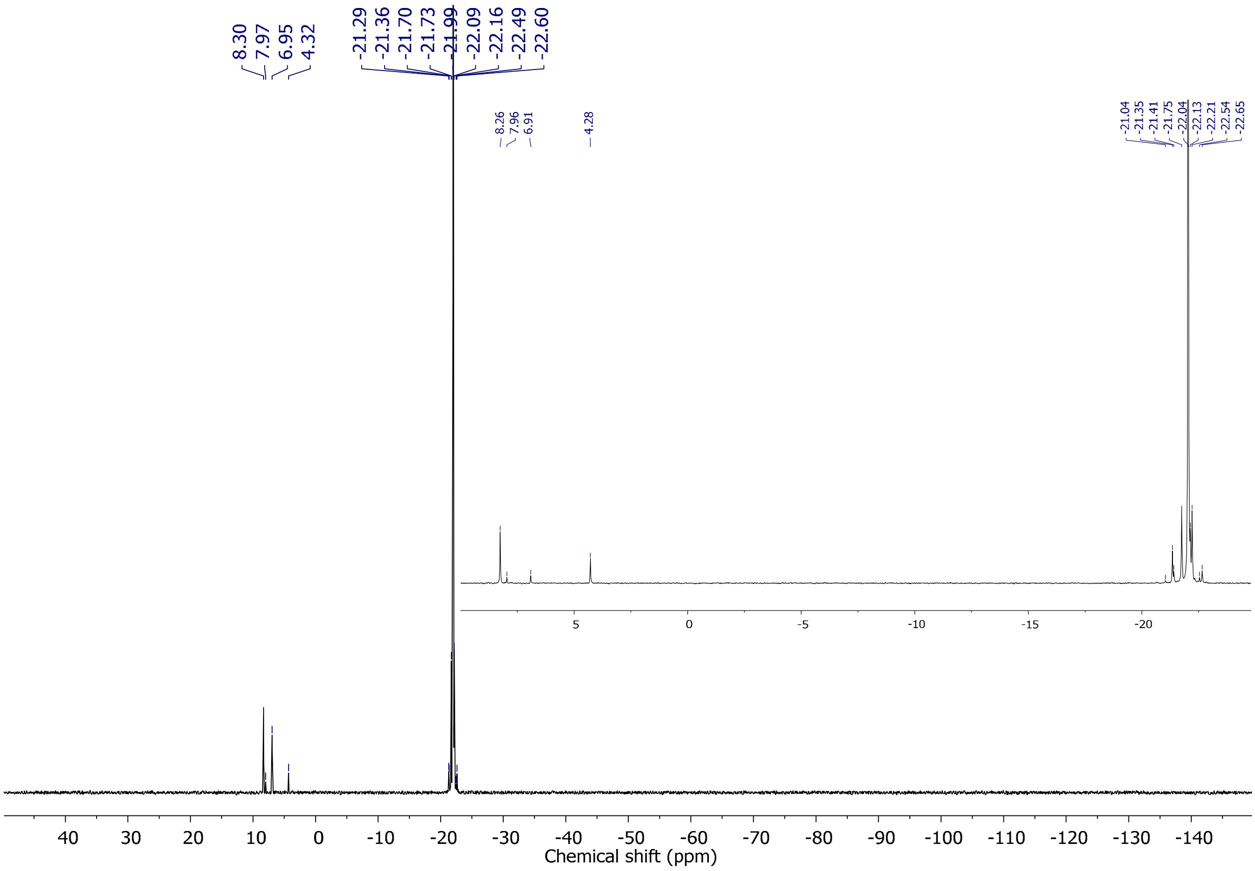

Figure S21. ^{29}Si NMR spectrum of reaction mixture C after reaction with an expanded view of the 10 to -25 ppm region.

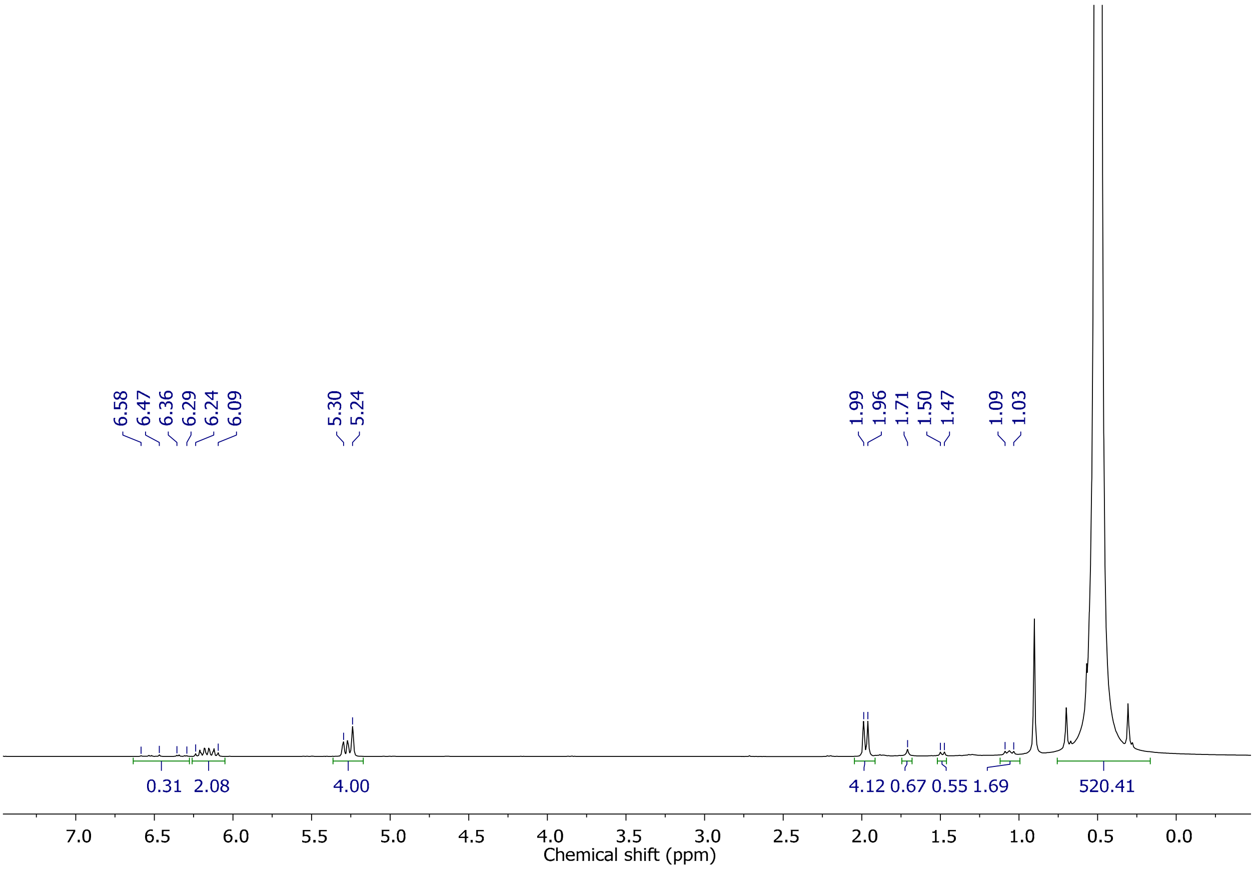

Figure S22. ^{1}H NMR spectrum of reaction mixture Bs after reaction.

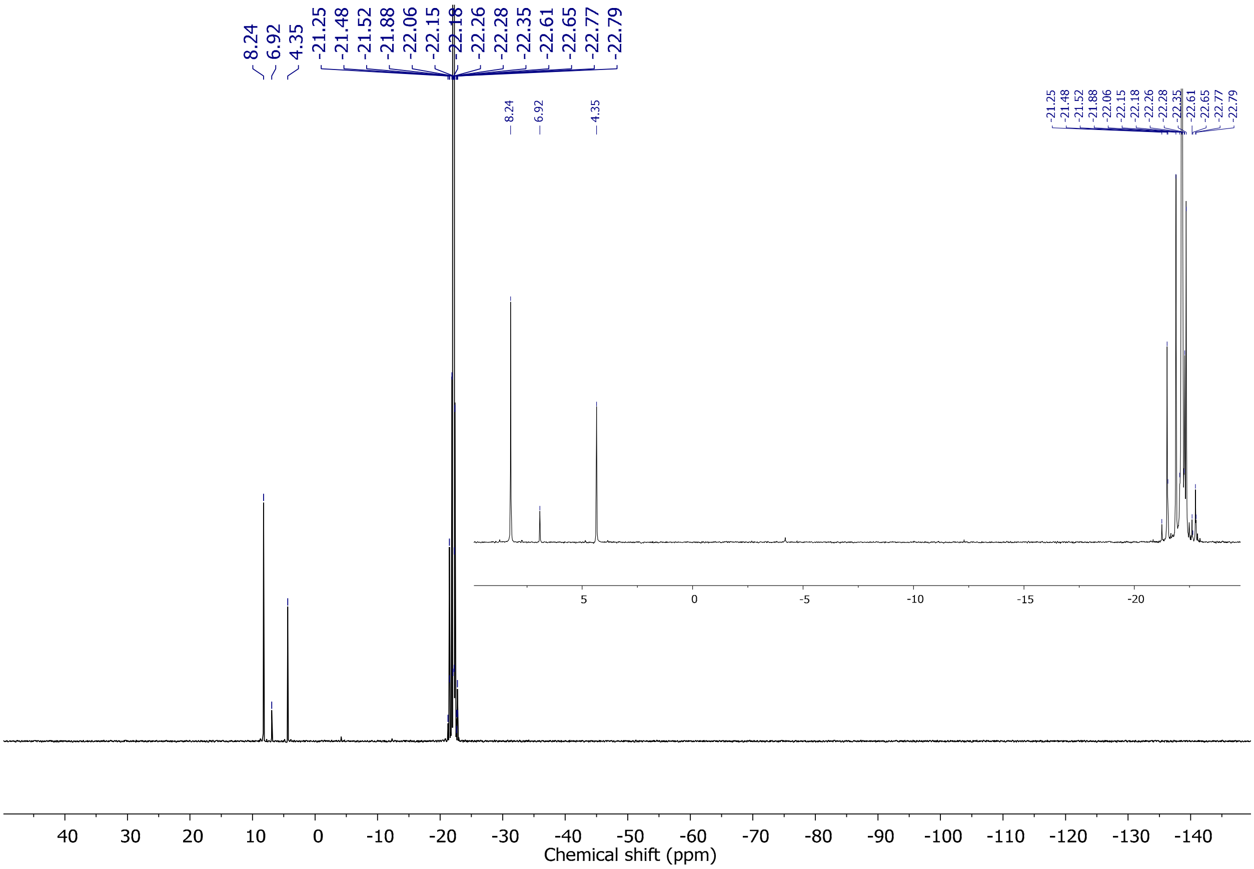

Figure S23. ^{29}Si NMR spectrum of reaction mixture Bs after reaction with an expanded view of the 10 to -25 ppm region.

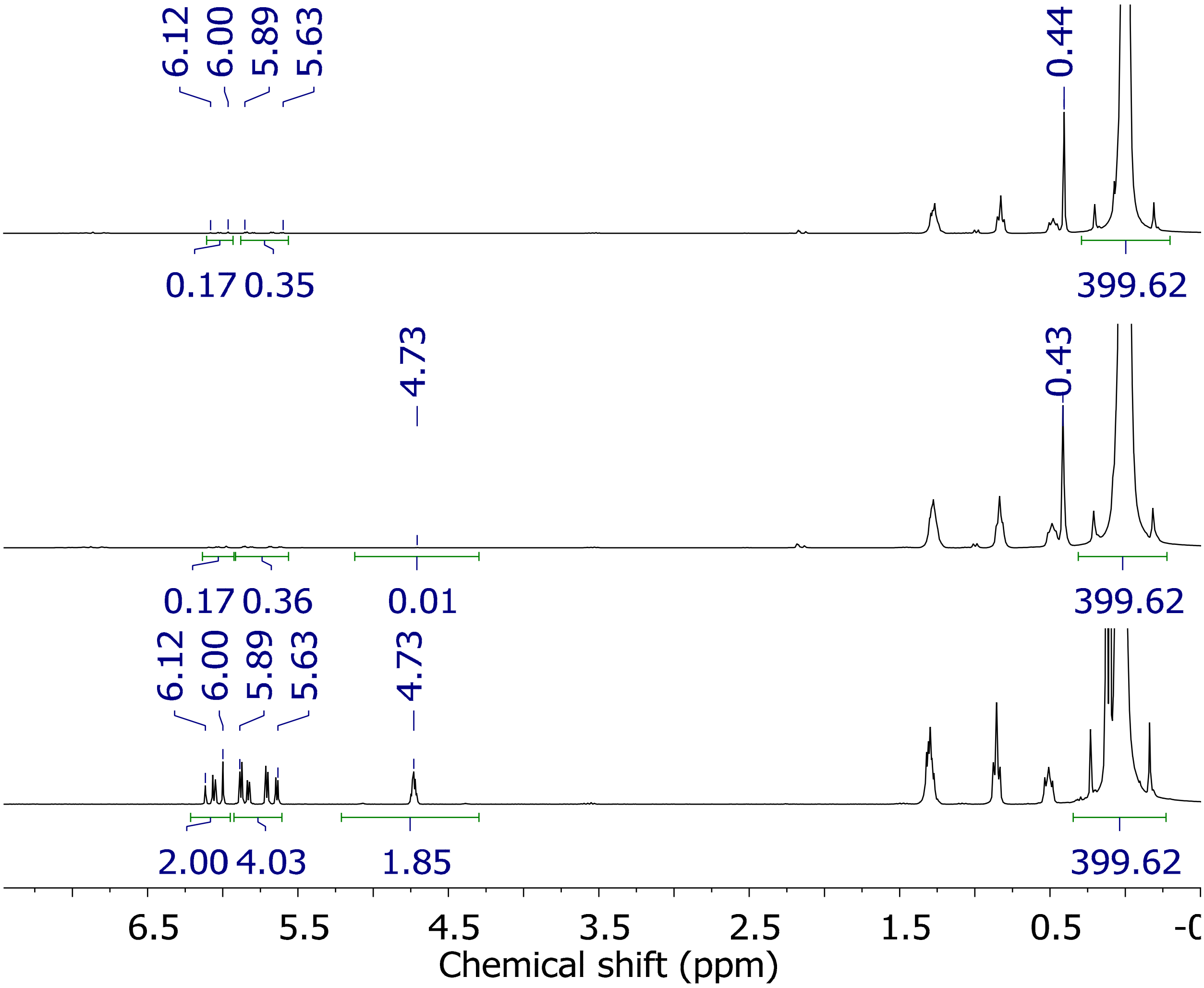

Figure S24. ^{1}H NMR spectra of reaction mixture D-vinyl.

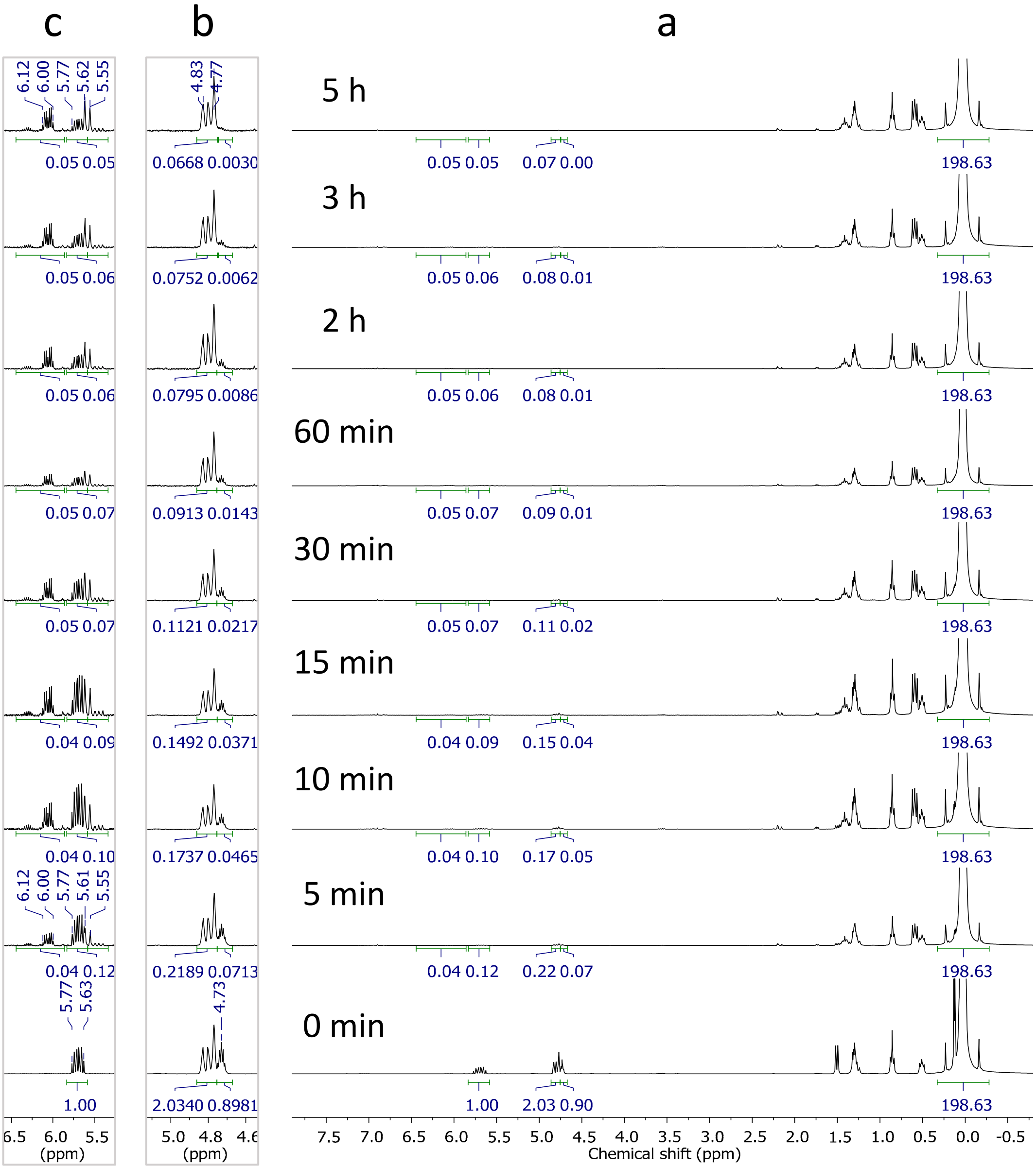

Figure S25. ^{1}H NMR spectra of reaction mixture D-allyl.

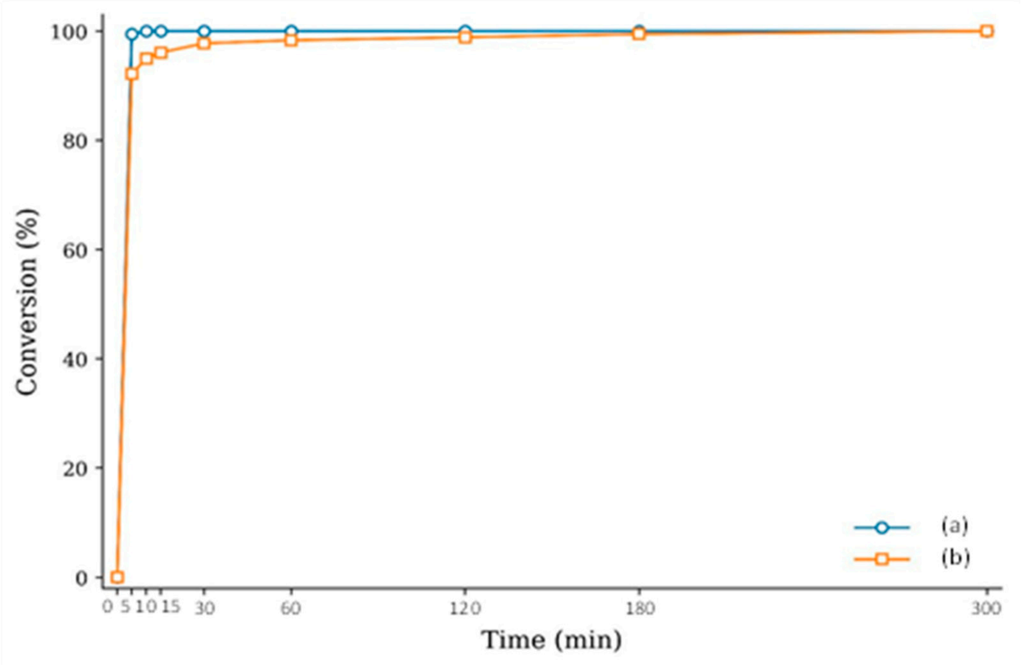

Figure S26. Experimental data for hydride functional groups vs time. (a) Conversion of hydride functional groups in reaction D-vinyl. (b) Conversion of hydride functional groups in reaction D-allyl.

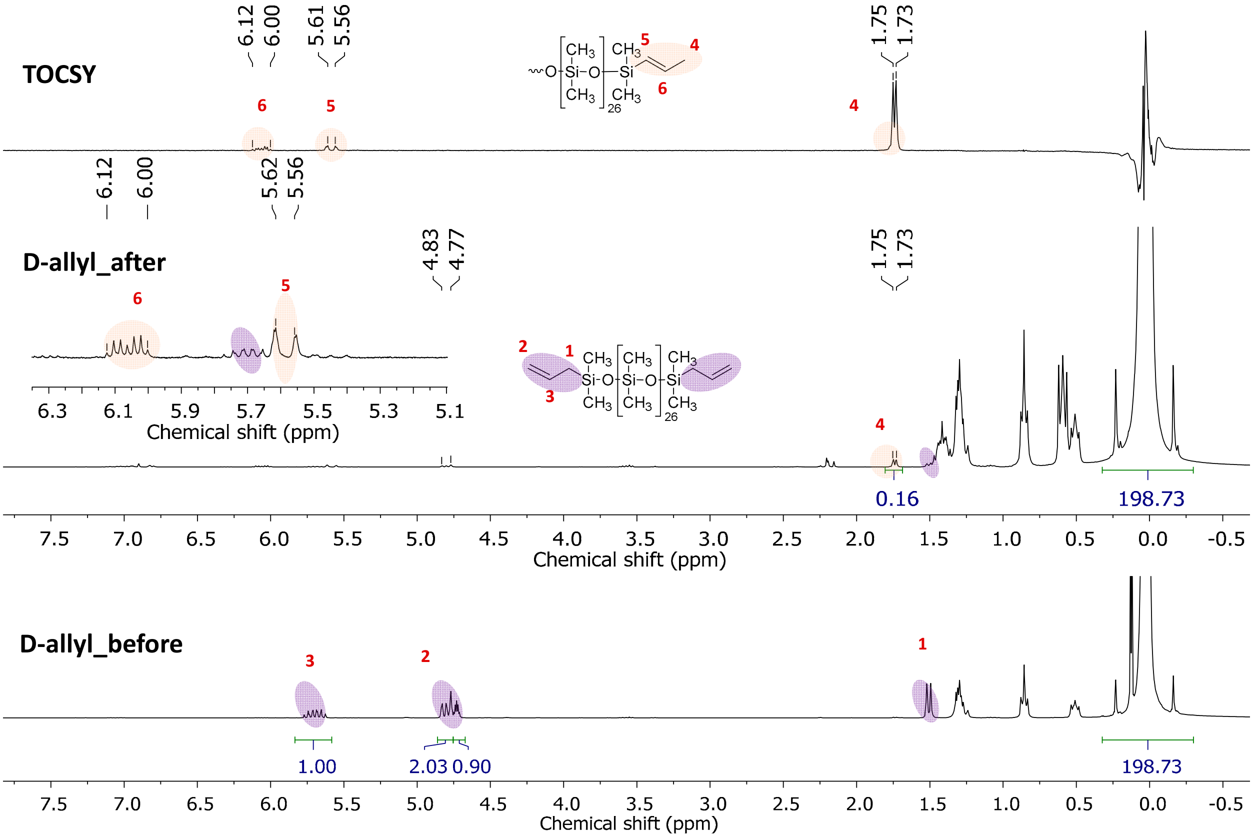

Figure S27 ^{1}H NMR spectra of the reaction mixture of reaction D-allyl before adding catalyst, after the reaction and one-dimensional total correlation spectroscopy.

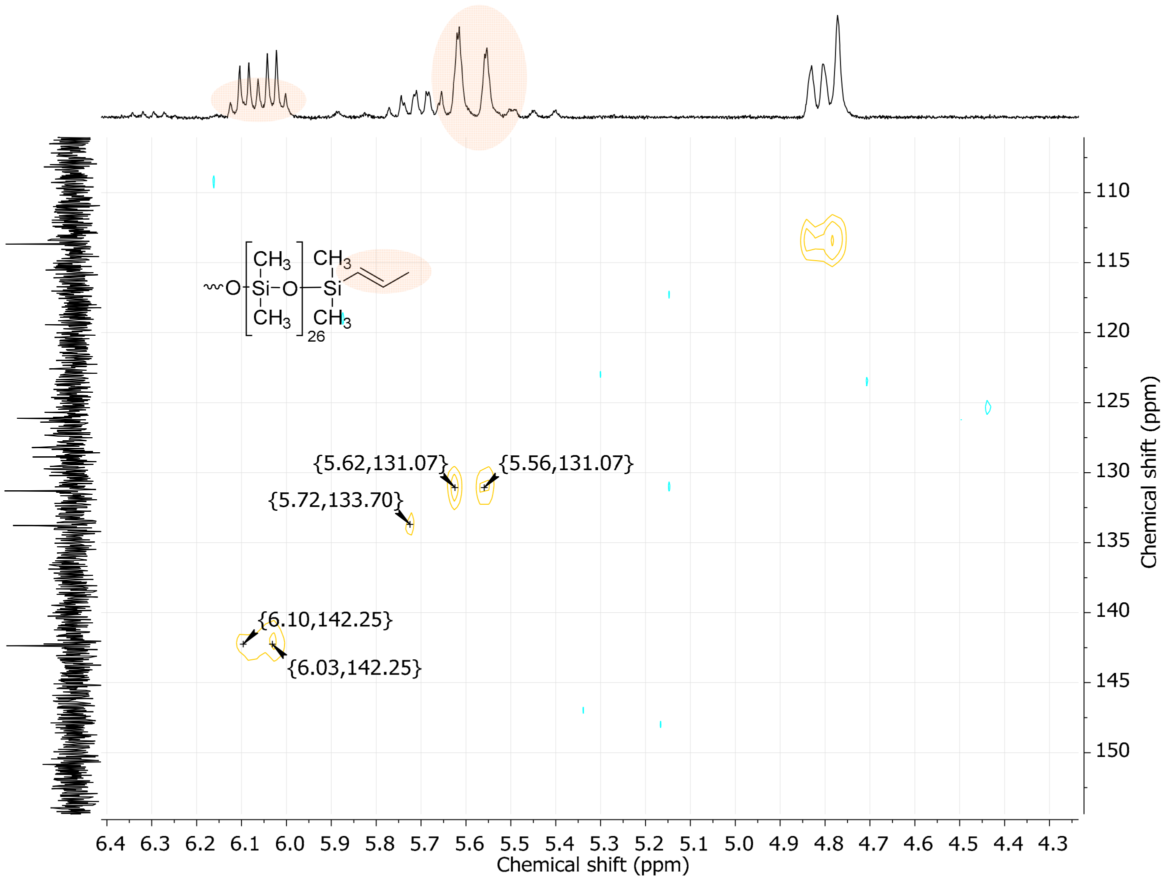

Figure S28 ^{1}H - ^{13}C HSQC NMR spectrum of the reaction mixture of reaction D-allyl.

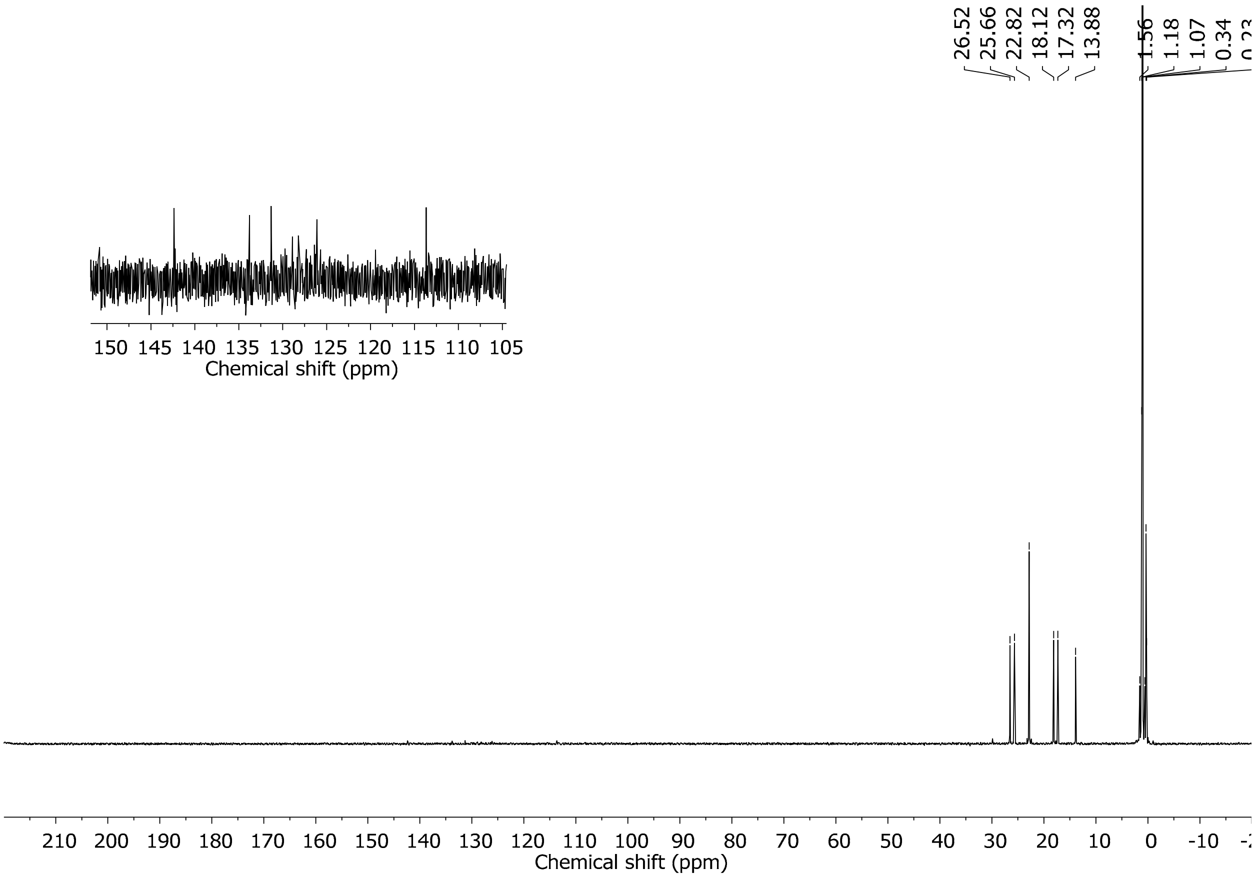

Figure S29 ^{13}C NMR spectrum of the reaction mixture of reaction D-allyl with an expanded view of the 150 to 105 ppm region.

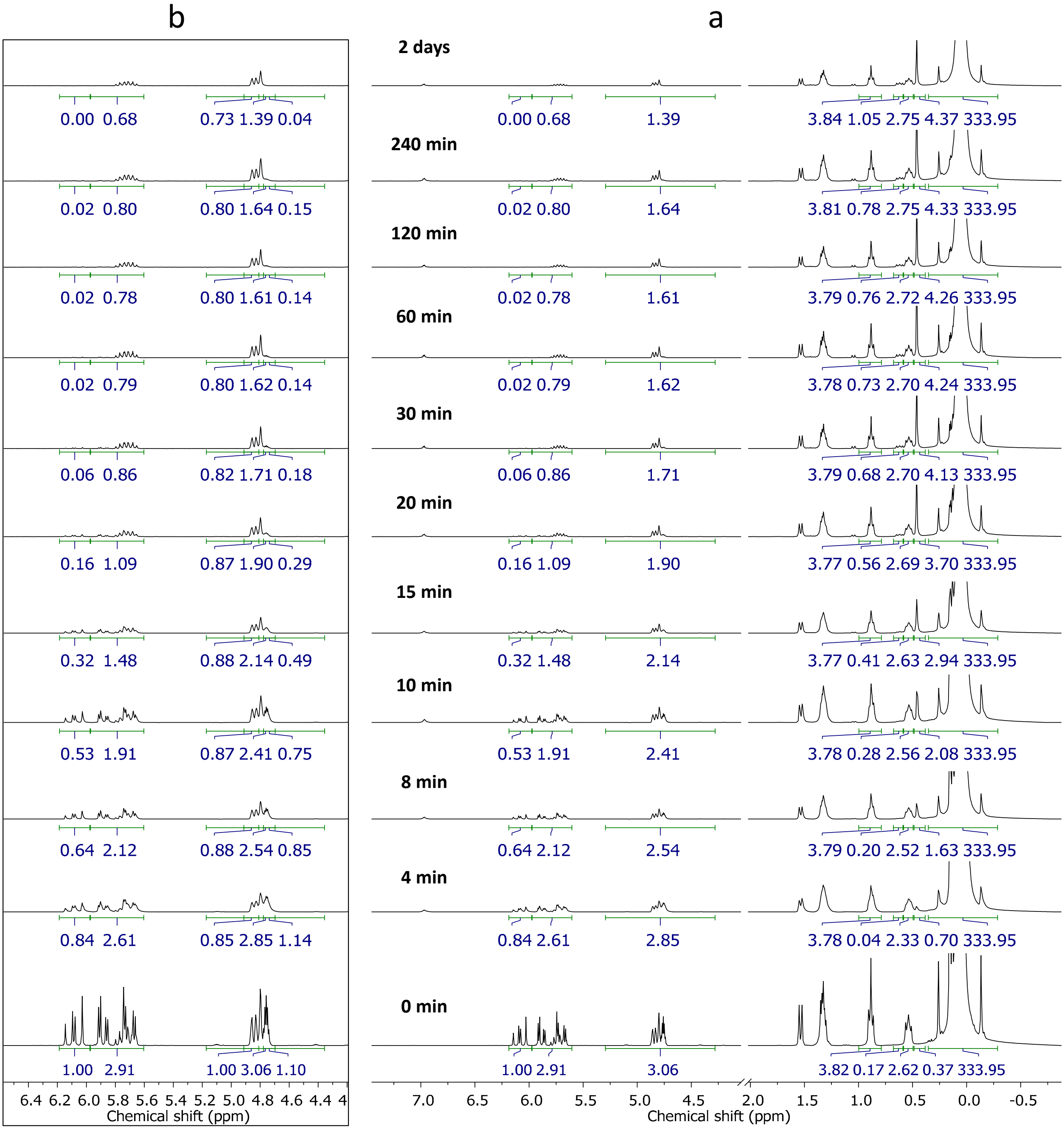

Figure S30. ^{1}H NMR spectra of reaction mixture E before adding catalyst and over a period of 2 days after catalyst addition (a) and with an expanded view of the 6.5 to 4.3 ppm region (b).

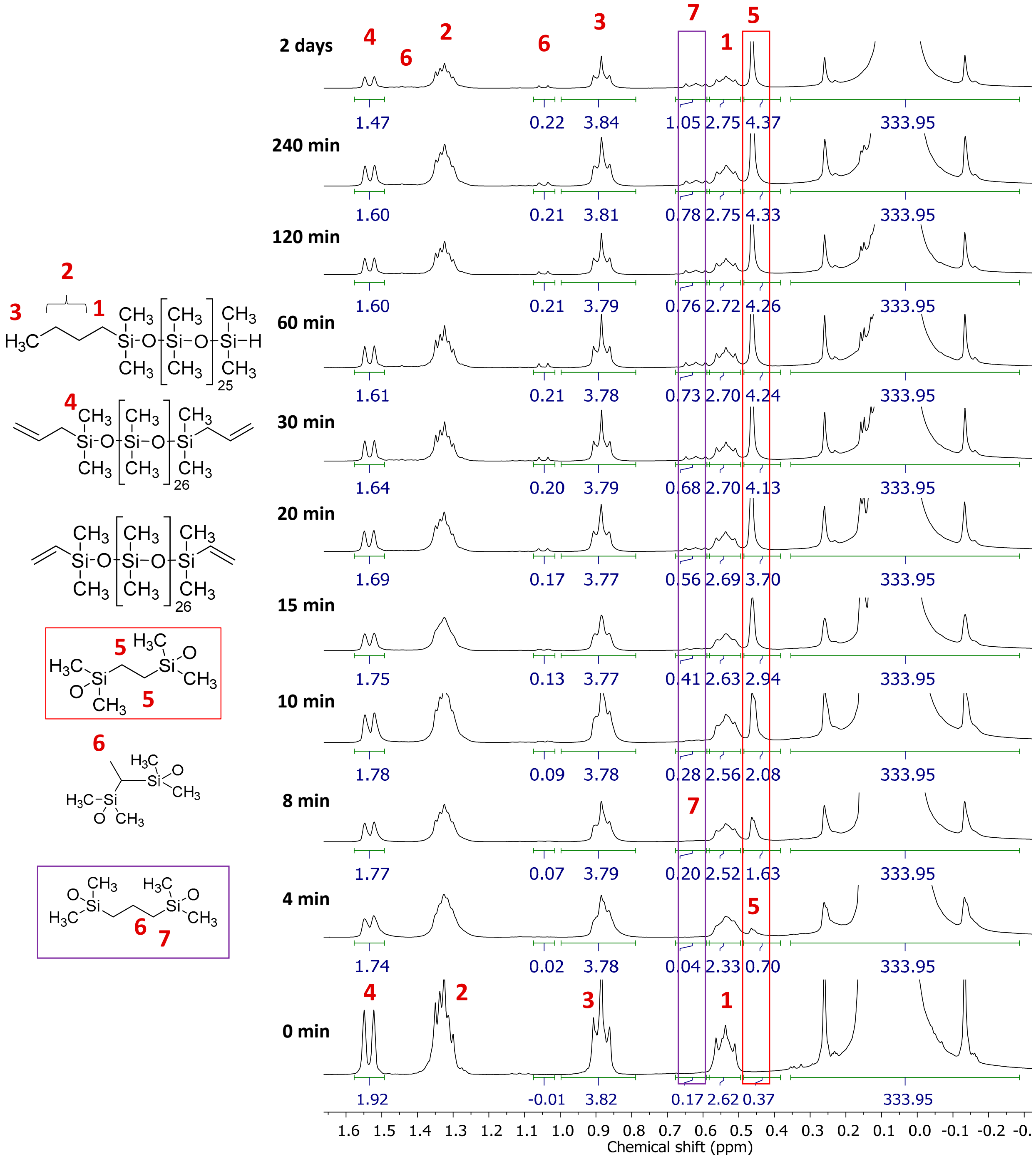

Figure S31. ^{1}H NMR spectra of reaction mixture E with view of the 1.6 to -0.3 ppm region.

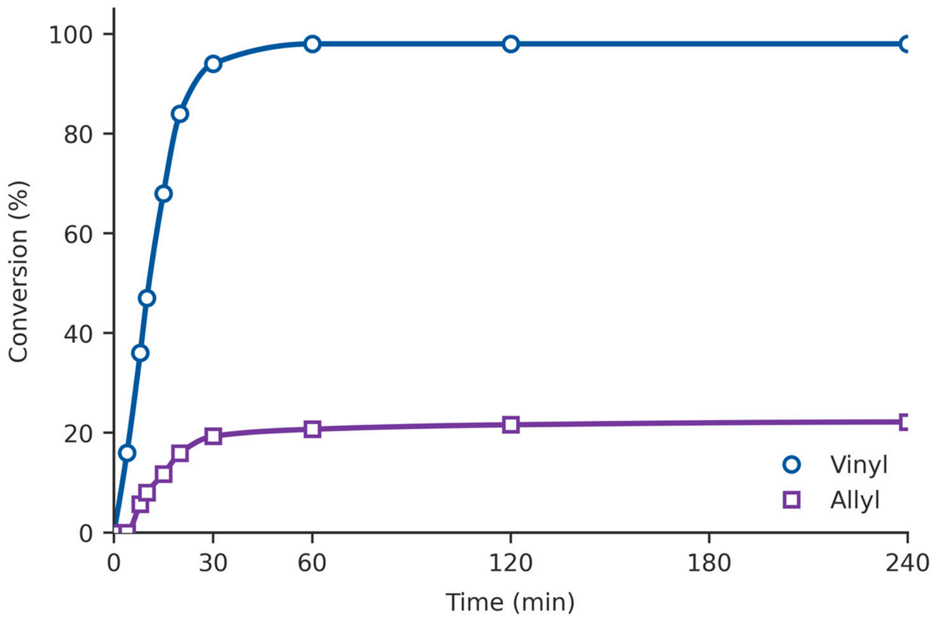

Figure S32 Conversion (%) of vinylsilyl and allylsilyl groups as a function of time (min).

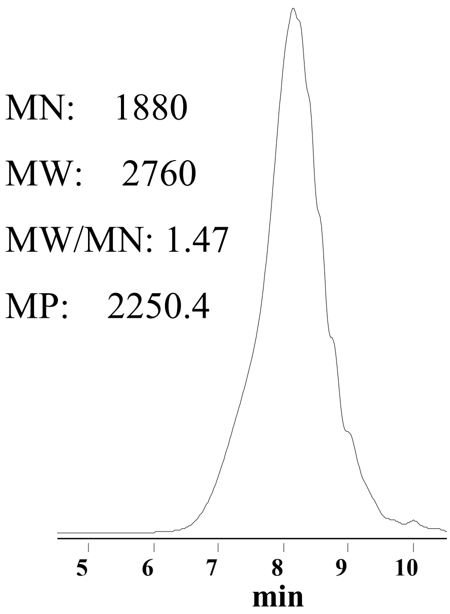

Figure S33. GPC curve of reaction mixture A before adding Karstedt’s catalyst.

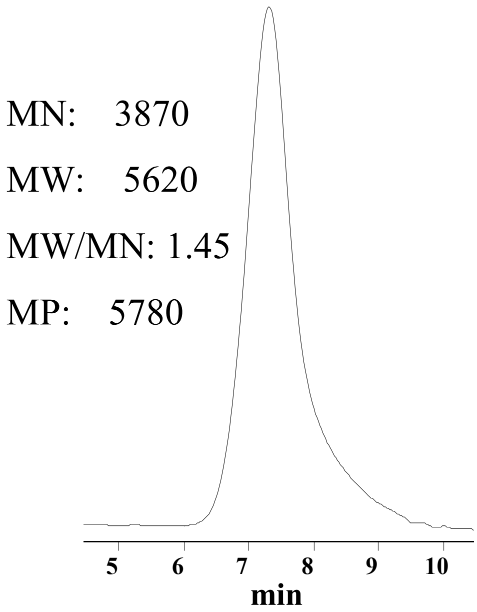

Figure S34. GPC curve of reaction mixture A after reaction.

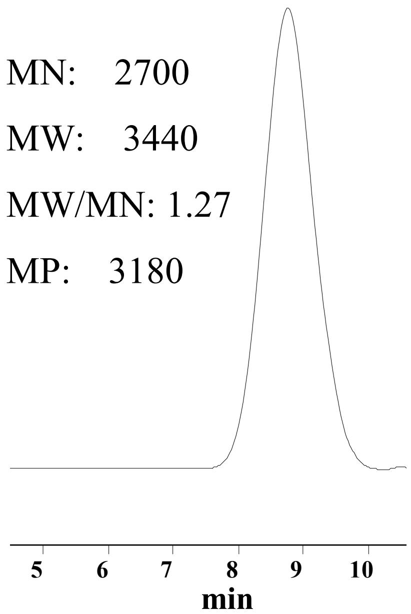

Figure S35. GPC curve of reaction mixture B before adding Karstedt's catalyst.

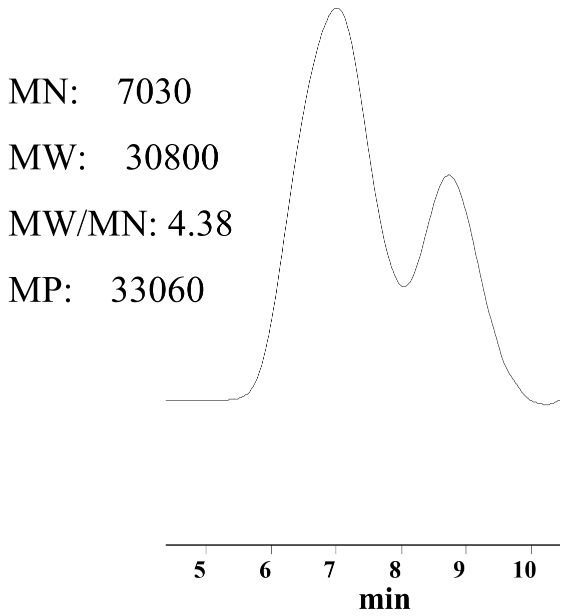

Figure S36. GPC curve of reaction mixture B after reaction.

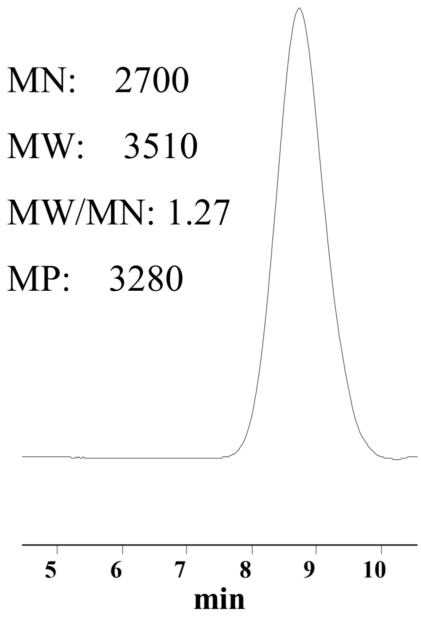

Figure S37. GPC curve of reaction mixture C before adding Karstedt's catalyst.

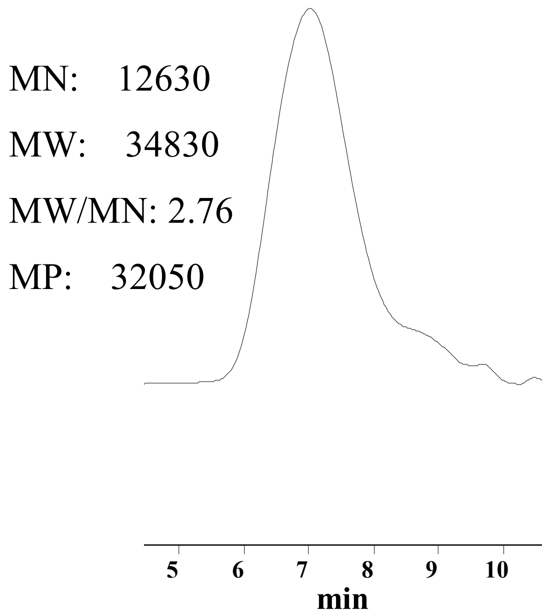

Figure S38. GPC curve of reaction mixture C after reaction.
